# Clean sweep or pathogen paradise? A critical review of cleaning and disinfection practices in broiler barns

**DOI:** 10.1016/j.psj.2025.106232

**Published:** 2025-12-11

**Authors:** Adrian Drögmöller, Helen Louton

**Affiliations:** a Animal Health and Animal Welfare, Faculty of Agricultural and Environmental Sciences, University of Rostock, Justus-von-Liebig-Weg 6b, Rostock D-18059, Germany; b Chair of Animal Welfare, Ethology, Animal Hygiene and Animal Husbandry, Department of Veterinary Sciences, Faculty of Veterinary Medicine, LMU Munich, Veterinärstr. 13/R, München D-80539, Germany

**Keywords:** Poultry, Disinfection, Cleaning, Method, Sanitation

## Abstract

Increased demand for broiler meat, coupled with increased consumer awareness and rising concerns about antimicrobial resistance, makes effective biosecurity more relevant than ever. The goal of this paper was to provide a systematic review of 77 published papers considering the effectiveness of cleaning and disinfection methods and antibacterial compounds applied in broiler barns and to present an overview of the current state of knowledge. To evaluate the completeness of methodological reporting, we developed an “information density” score based on factors described as relevant for the effectiveness of cleaning and disinfection. Aldehydes and quaternary ammonium compounds were the most researched disinfectant compounds, appearing in 57.14% and 48.05% of the papers, respectively. Very few papers presented an adequate amount of data about the cleaning and disinfection procedures, several did not mention the used compounds, and most papers did not describe temperature, detergent use, waiting time, or application technique. The average information density of the papers presenting on-site studies was 44.81%. Based on available numeric data, disinfectants containing aldehydes were overall the most effective disinfectants across all bacteria followed by peroxides, QACs and organic acids, whereas those with potassium peroxymonosulfate or iodine performed the worst. Furthermore, we found a difference in compound effectiveness between relevant pathogens, e.g., QAC-based disinfectants were not as effective against *Staphylococcus* as against other pathogens. In addition, disinfection effectiveness can be reduced by the presence of organic material, and some risk factors regarding the used water require further investigation to yield conclusive results. The majority of the reviewed studies did not provide enough methodological detail. Critical parameters including water temperature, application technique, detergent use, exposure time, and surface type were often incompletely reported or entirely missing. As a result, the calculated “effectiveness” scores may partly reflect differences in study design and reporting quality rather than the true antimicrobial activity of the disinfectants. Overall, more transparency about the cleaning and disinfection methods is needed to gain knowledge about effective measures against bacterial pathogens.

## Introduction

Broiler meat is currently the most important type of meat to be produced. Between 2002 and 2022, the global production of poultry meat increased by 87%, amounting to 139.2 million metric tons of meat, surpassing all other meats (Statistisches Bundesamt, 2024). Furthermore, the number of slaughtered chickens reached 26.6 billion in 2022, a 68% increase compared with 2002. Broiler chickens have a relatively good feed conversion ratio (Mekonnen et al., 2019) and low greenhouse gas emissions compared with other meat-producing animals (Clune et al., 2017), making broiler meat an economically and ecologically viable option. The relatively young slaughter age of broilers allows for multiple production cycles per year and thus likely minimizes monetary risk (Baéza at al., 2012). Additionally, the persistent use of chicken meat in cuisines around the world, with little to no religious taboos, paired with the global increase in economic wealth, has led to an increase in consumption and production (Nam et al., 2010).

At the same time, consumers perceive antibiotic use as critical and thus increasingly demand antibiotic-free chicken meat (Haque et al., 2020). Additionally, antibiotic-resistant pathogens have been observed in broiler chicken production (Mehdi et al., 2018). Therefore, measures to reduce antibiotic use are necessary. Despite the decreasing antibiotic sales per population correction unit in the EU, a huge difference remains between countries, indicating a potential lack of effective prophylactic measures in countries with high antibiotic use (European Environment Agency, 2024). While breeding broilers with a stronger immune system is one possible option, another approach is the examination and modification of biosecurity measures. Cleaning and disinfection of the barn prior to introducing new chicks play a key role in preventing a carry-over of pathogens from the previous flock while also reducing the initial infection pressure (Luyckx et al., 2015a, 2015b). Since 2007, disinfection of broiler barns after final depopulation and before introduction of new chicks has been common practice in the EU according to Council Directive 2007/43/EC (Council of the European Union, 2007). Even though Germany is known for strict animal welfare laws, very little specific requirements regarding disinfection and cleaning are ever stated in German laws. The most detailed description can be found in the regulation on the control of *Salmonella* in domestic chickens and turkeys (Geflügel-Salmonellen-Verordnung – GflSalmoV), which states that in case of *Salmonella* infection, the barns should be cleaned, disinfected, and measures against pathogens should be commenced while the success should be confirmed with swabs or plate counts (Bundesamt für Justiz, 2009). It is left open who should perform said measures, no indication of needed certification or training is given, it is not explained how the sampling should work nor are steps for successful disinfection mentioned. A document closest to an official guideline can be found in the “Guide to good hygiene practice for the prevention and control of pathogenic microorganisms with particular reference to *Salmonella* in *Gallus gallus* (broilers) reared for meat—on farms, and during catching, loading and transport” or short, the “European Poultrymeat Industry Guide” (A.V.E.C. and COPA-COGECA, 2010). The guide recommends the following steps: soaking at low pressure, washing with detergent at high pressure, spraying with disinfectant, and additional fogging. Those guidelines are seemingly still somewhat followed in Germany (Mateus-Vargas et al., 2022). However, research conducted after publication of the guide has shown that fogging alone is an effective disinfection method and does not necessarily need to be preceded by spraying of disinfectant (Luyckx et al., 2015b; Battersby et al., 2017). The “European Poultrymeat Industry Guide” furthermore provides a detailed plan for sampling, which includes the use of large fabric swabs and 10 different samples per location category (e.g., air inlets, walls, drinkers). Whereas the sampled locations are mostly consistent, the sample size is usually smaller than recommended and other sample techniques, such as wipes or boot swaps, are used in field research (e.g., Tessin at al., 2024).

Despite those clear recommendations, cleaning and disinfection protocols in broiler barns remain inconsistent and often poorly documented in practice. Several studies across a long time span have reported positive pathogen detections even after thorough sanitation measures (Higgings et al., 1982; Mateus-Vargas et al., 2022), highlighting a potential gap between expected efficacy and field performance. This discrepancy raises important questions about the effectiveness of commonly used disinfectants, the influence of external factors such as organic matter or water quality, and the lack of standardization in disinfection protocols. Furthermore, the disinfection procedure is a very delicate process. Factors such as the disinfectant compounds, number of disinfectants, use of cleaning products, temperature, exposure time, and husbandry system as well as interactions between these factors influence the overall success rate (Maertens et al., 2018). Despite contractors performing better than farmers (Maertens et al., 2018), hiring professionals and using extensive supplies and techniques is an economic decision not every farmer can easily make.

This review aims to bridge that gap by systematically evaluating the effectiveness of cleaning and disinfection procedures in broiler production, with a focus on both the types of agents used and the methodological rigor of their application. By compiling and critically analyzing available data, this paper seeks to identify effective compounds against key pathogens, evaluate gaps in procedural transparency, and provide targeted recommendations to enhance biosecurity. The overarching goal is to inform practitioners, reduce reliance on antibiotics, and ultimately improve animal health outcomes and food safety.

## Materials and methods

### Literature search

**Fig. 1:**
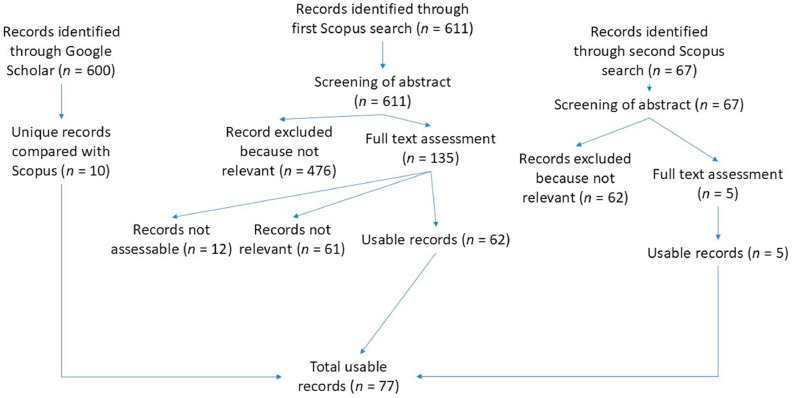
Flow diagram illustrating the literature search and selection process for studies on cleaning and disinfection in broiler barns. Records were identified through Scopus and Google Scholar, screened for relevance, and assessed for eligibility. In total, 77 usable records were included in the final synthesis. Percentages and exclusion numbers refer to the total number of records screened.

The main part of the literature research was conducted from August 3rd to November 18th, 2024, excluding papers from 2024. A systematic search was performed in the Scopus database by using the following search string: “TITLE-ABS-KEY (broiler AND disinfection OR disinfectant) OR TITLE-ABS-KEY (broiler AND sanitation OR sanitizer) OR TITLE-ABS-KEY (broiler AND cleaning OR cleaner).” All papers published before 2024 were included in the search. The search results were screened for relevance based on title and abstract, followed by full-text assessment. Records not relevant to disinfection in broiler barns were excluded. In addition, 10 unique records from Google Scholar that were not indexed in Scopus were included. Fig. 1 provides an overview of the identification, screening, exclusion, and inclusion process according to PRISMA guidelines (Page et al., 2021). In the first step, the title and abstract were used to group relevant papers in broad categories such as “*in vitro* tests,” “general cleaning,” “unclear,” and “not relevant” by using Zotero software (Corporation for Digital Scholarship, Vienna, VA). For inclusion in our analysis, studies involving *in vitro* testing were required to use bacterial isolates originating from broiler farms or to have obtained these isolates directly on site. Field studies had to be conducted on broiler farms, not in hatcheries, transport crates, or processing facilities. After narrowing down the focus to just cleaning and disinfection, all papers remaining relevant or unclear (*n* = 135) were thoroughly read and either used or excluded. During that process, an additional paper was found among the references listed in another one. In total, 62 papers were found, excluding 12 that could not be used owing to the lack of access or not being found besides their entry. In March of 2025, from the 5th to the 28th, the same search string was used to find all new papers from 2024. Of the 67 new ones, 5 could be used, resulting in a total of 67 papers from Scopus.

Additionally, a Google Scholar (Alphabet Inc., Mountain View, CA) search was used to gather additional material with the search term “broiler disinfection” during the same timeframes in 2024 and 2025. There, the first 600 search results were screened. Articles with relevant content were read and compared with the existing sources. This search resulted in 10 additional papers, totaling the papers included in this review to 77.

To ensure the reliability of the extracted information, a basic appraisal of study quality was performed. This appraisal considered (i) the completeness and clarity of the disinfection description, (ii) the experimental design and methodological transparency, and (iii) the reporting of microbial outcomes. Studies with insufficient methodological detail or unclear results were categorized as “not assessable” and excluded from further analysis.

### Data processing

After data collection in Zotero, the data was transferred to Microsoft Excel (Microsoft Company, Redmond, WA) for further processing. To each paper, the publication year, author country of origin, considered pathogen, and used disinfectant compound, if available, were noted. The next step was to examine the “research goal,” which was defined as either directly looking into the effect of (cleaning and) disinfectant compounds or other goals such as risk factor analysis or epidemiological survey. Then, the kind of disinfectant compound, i.e., new/experimental or commercially available, and the location, i.e., barn or laboratory, was added alongside a remark if only disinfectant (or cleaning) compounds or the whole cleaning process was analyzed in the papers.

To evaluate the completeness of methodological reporting, an “information density” score was developed based on factors described as relevant for the effectiveness of cleaning and disinfection in poultry houses (A.V.E.C. and COPA-COGECA, 2010; Maertens et al., 2018). Seven categories were assessed: (1) mention of a cleaning step, (2) use of a detergent, (3) cleaning method (application), (4) water temperature, (5) time between cleaning and disinfection, (6) disinfection method, and (7) disinfectant compound. Each category was coded as 1 (information provided) or 0 (no information). The sum was divided by the number of categories and expressed as a percentage (“information density”). This score is intended to describe the completeness of methodological information rather than experimental quality. Although the score has not been validated formally, it is based on transparent, literature-derived criteria and can therefore be reproduced for future comparative analyses. The criteria included in the “information density” score and their coding scheme are summarized in Table 1 to enhance transparency and reproducibility of the scoring process. *In vitro* papers were excluded because they did not involve a cleaning procedure and would therefore artificially have reduced the overall information density. Specific data such as “pathogens” and “compounds” were collected in separate “paper-pathogens” and “paper-compounds” tables to ease the calculations.

**Table 1:** Criteria included in the calculation of the “information density” score.

Category	Description	Coding (binary)	Example of information considered as “present”
1. Cleaning step	Mention of a cleaning procedure prior to disinfection	1 = yes; 0 = no	“The barn was cleaned before disinfection.”
2. Detergent use	Use of a detergent or cleaning agent explicitly stated	1 = yes; 0 = no	“A detergent-based foam cleaner was applied.”
3. Cleaning method	Description of the cleaning method used	1 = yes; 0 = no	“High-pressure water cleaning” or “manual scrubbing”
4. Water temperature	Indication of water temperature during cleaning	1 = yes; 0 = no	“Water at 60°C was used for washing.”
5. Time between cleaning and disinfection	Time interval specified or implied	1 = yes; 0 = no	“Disinfection was carried out 24 h after cleaning.”
6. Disinfection method	Description of application method	1 = yes; 0 = no	“Spraying,” “fogging,” or “foam application”
7. Disinfectant compound	Active substance(s) or product type mentioned	1 = yes; 0 = no	“Glutaraldehyde–formaldehyde mix,” “peracetic acid”

### Calculation and recommendations

To present the best results, 2 kinds of analysis and presentation were chosen. The first is a more qualitative approach, in which the presented data is handpicked and presented under all relevant factors mentioned in that study. The most relevant information can be found in the Results section, and a more detailed overview is available as supplementary material. A qualitative analysis of the available data proved challenging. The absence of standardized disinfection protocols, the use of heterogeneous performance indicators, and, most importantly, the lack of transparent descriptions of disinfection procedures precluded a proper meta-analysis for nearly all pathogens, because fewer than 5 comparable studies were available in most cases. Furthermore, reported outcomes of disinfection efficacy or effectiveness varied considerably among studies, including log-reductions in bacterial counts, colony-forming units per surface area, and prevalence data from environmental samples. Because these endpoints were not directly comparable, no formal meta-analysis was conducted. Instead, “average effectiveness” was derived from a qualitative synthesis of the reported results. To incorporate all available evidence and minimize potential reporting bias, a ranking-based approach, rather than a quantitative synthesis, was adopted to assess disinfection effectiveness. Although statistical pooling was not feasible, each study was treated as an individual application trial. All studies providing sufficient information on disinfection efficacy or effectiveness were included, provided that overall effectiveness—independent of specific performance indicators—was clearly reported. Studies were excluded if total effectiveness was ambiguous, for instance if only partial inhibition rates (e.g., 95% microbial inhibition) were presented.

A 3-level ranking system was developed to evaluate disinfection performance. *In vitro* assays served as the foundation for determining baseline efficacy against the target pathogens. Compounds demonstrating complete efficacy in all *in vitro* tests were assigned a “+”. Lower but still notable ≥99.99 efficacy was denoted as “.”, whereas insufficient outcomes received a “−”. The same classification was applied to *in vitro* tests performed in the presence of organic matter.

For on-site evaluations, ranking was based on reported field performance. A “+” was assigned if at least 1 study achieved complete disinfection under practical conditions; a “.” indicated the highest reported effectiveness to be ≥99.99%, and a “−” was assigned for lower performance levels. General performance was further assessed based on the proportion of studies achieving specific thresholds: a “+” was given if more than half of the studies reported ≥99.99% effectiveness, a “.” if more than half reported ≥90.00%, and a “−” otherwise.

Compounds receiving predominantly or exclusively “+” ratings were considered highly effective and, depending on the number of independent sources, likely robust across varying experimental conditions. A “.” indicated moderate or condition-dependent efficacy or effectiveness. This ranking framework thus enabled the identification of consistently effective disinfectants despite the high variability among studies, while also providing a transparent rationale for the inclusion, interpretation, and recommendation of specific compounds.

Disinfection performance data were categorized according to test level, distinguishing between *in vitro* tests (with or without organic load) and field trials conducted under commercial conditions. Where information on organic load was available, results were reported separately. However, owing to the limited number of comparable studies within each test level, no formal stratified statistical analysis was possible.

Owing to the substantial heterogeneity in study designs, outcome measures, and reporting formats, a quantitative meta-analysis with calculation of confidence intervals or heterogeneity statistics (I²) was not feasible. Many studies lacked standardized effect measurements or variance data. Instead, a descriptive vote-counting approach was applied to indicate how consistently specific disinfectants were reported as effective across independent studies. The present synthesis focused on a transparent qualitative ranking approach, which provides an overview of consistent trends.

Although an economic comparison of disinfectants (e.g., cost per square meter, labor time) was originally intended, it is beyond the scope of this review owing to the lack of available data. Reliable cost data were rarely reported and are highly region dependent, influenced by factors such as national regulations under the EU Biocidal Products Regulation (Regulation [EU] No 528/2012; European Parliament and Council, 2012), product availability, and market and labor prices. As these parameters also change over time, direct comparisons would not be meaningful. Whenever possible, future research should integrate effectiveness and cost considerations under standardized, region-specific conditions to support evidence-based decisions in poultry production.

## Results and discussion

### General

In total, our analysis included 77 papers presenting results regarding the cleaning or disinfection or both of broiler barns or the disinfection of bacterial isolates from said barns. Of those papers, 67 were found via Scopus, whereas 10 were exclusively found via Google Scholar. The median release year was 2016. The timeframes 2024 to 2020, 2019 to 2010, and before 2010 (i.e., 1982 to 2009) were equally represented, with 26, 25, and 26 papers, respectively, which might be explained by an ever-increasing demand for chicken meat (Food and Agriculture Organization of the United Nations – with major processing by Our World in Data, 2025) and therefore increased research.

Although reducing the stocking density or the number of barns per farm could reduce the risk of disease outbreaks (EFSA Panel on Biological Hazards et al., 2019) and therefore antibiotic usage, such measures would pose a serious risk for food security and the economic viability of poultry farms. Therefore, the focus should be on biosecurity, including cleaning and disinfection. The heightened research interest in disinfection methods following 2020 could be due to the shift in perspective on antibiotic resistance in pathogens mentioned by Podolsky (2018). Specifically, it could originate from a public health–focused interest in preventing the development of antibiotic resistance by reducing antibiotic use because the bacteria species present in broiler barns are partially the same kind as multi-resistant pathogen strains found in hospitals (Church and McKillip, 2021). Furthermore, *Salmonella*, a pathogen commonly associated with poultry, has notable resistance against certain antibiotics (Abd El-Aziz et al., 2021), giving prevention through biosecurity a high priority.

The most prominent country in our dataset was Egypt, with 14 papers originating from there, followed by France, Germany, the UK, the USA, and Brazil with 5 papers each. Egypt’s broiler production as seen in the “Our World in Data” dataset and the year of publication revealed an interesting pattern: The publishing dates fell into periods of increased production of chicken meat. Following 2016, chicken meat production increased by 250%, explaining a heightened interest in the cleaning and disinfection of barns. Furthermore, the EU Member States (excluding Great Britain) published 29 papers, with a relatively even split between the previously mentioned timeframes: 9 papers from 2024 to 2020, 10 papers from 2019 to 2010, and 10 papers before 2010. The increased number of publications toward the present might be explained by the EU zero pollution action plan, which calls for a massive reduction in antibiotic sales (European Environment Agency, 2024) and thus makes information regarding effective biosecurity increasingly important.

**Fig. 2:**
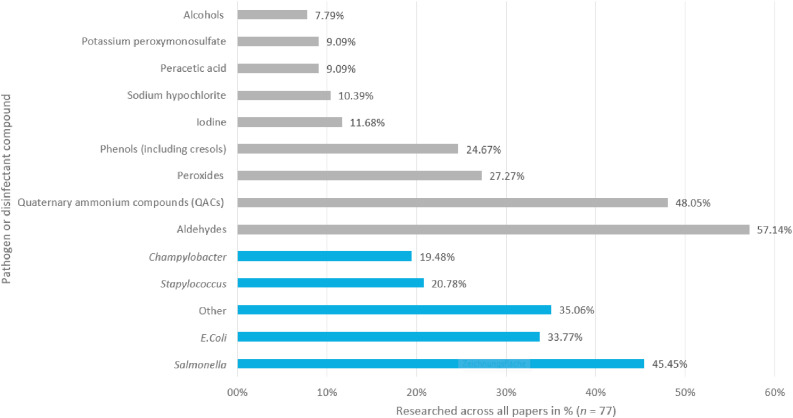
Researched pathogens and disinfectant compounds in studies on cleaning and disinfection in broiler barns (*n* = 77).

We furthermore found a near-even split in the research focus, with 33 papers dealing with cleaning or disinfection or both and 34 papers dealing with other topics such as general risk factors or the persistence of pathogens. As seen in Fig. 2, the most researched pathogen was *Salmonella*, appearing in 35 papers (45.45% of all papers), followed by *Escherichia coli* with 26, *Staphylococcus* with 16, and *Campylobacter* with 15 papers. Other pathogens, including but not limited to unspecified bacteria, *Enterococcus*, and *Streptococcus*, were reported in 27 papers. The investigated disinfectant compounds varied greatly. The most frequent ones were aldehydes (used in 82 disinfectants and investigated in 57.14% of the papers), quaternary ammonium compounds (**QACs**) (69), peroxides (29), phenol (including cresols) (24), alcohols (11), sodium hypochlorite (10), iodine (9), peracetic acid (7), and potassium peroxymonosulfate (7) (Fig. 2).

**Fig. 3:**
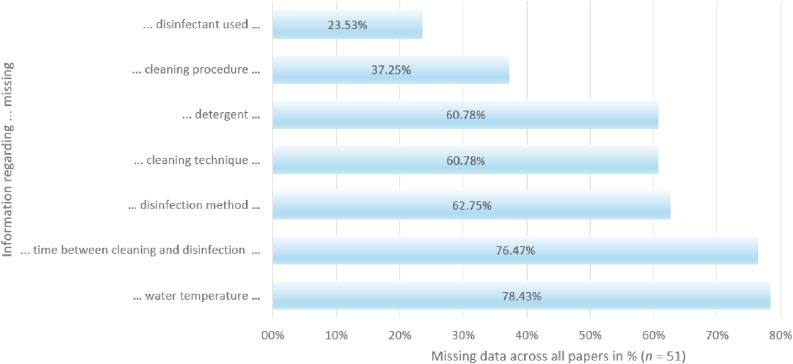
Amount of missing data in relevant categories regarding the cleaning and disinfection of broiler barns (*n* = 51).

The average information density of the papers presenting on-site field research was 44.08%. *In vitro* papers were excluded from the calculation of average information density because they did not include a cleaning procedure (as mentioned in the Materials and Methods section). As seen in Fig. 3, across all applicable papers (*n* = 51), 40 did not mention the water temperature during cleaning, 39 did not specify the time interval between cleaning and disinfection, 32 failed to mention the disinfection method, 31 did not describe the cleaning technique, 31 did not mention the use or non-use of detergents, 19 gave no indication what cleaning procedure was used, and 12 even failed to specify the disinfectant.

If the information was present, most cleaning was done with a combination of wet and dry cleaning and with a pressure washer. Cold water was used in 8 papers, warm water in 9. In 23.52% of the papers, the detergent was specified, which in 25.00% of the cases was sodium hydroxide. Most commonly, disinfection was done 24 h after cleaning. Fogging was used in 19.23% of the papers, spraying of the disinfectant in 25.00%.

As seen in Table 2, most disinfectants were generally effective against the pathogens investigated *in vitro*. Notable exceptions were iodine and potassium peroxymonosulfate, both of which only selectively achieved full disinfection even in the absence of organic matter. The general trend of most disinfectants to perform worse when organic matter was present during *in vitro* testing highlights the need for proper cleaning. Overall, as seen in Table 3, aldehydes and QACs were the most effective disinfectants on site, whereas potassium peroxymonosulfate and iodine were found to be ineffective. As seen most notably with the QACs, some studies succeeded in complete disinfection, but most did not. These findings strongly suggest that external factors such as cleanliness (if considering Table 2) or other risk factors might be influencing the results. However, differences in sample locations and protocols might have prevented localized detection, highlighting the need for standardized testing practices and transparent data presentation to allow identification of those critical factors. Furthermore, the impact of biofilms, local genotypes, and potentially different strains of the same class of bacteria could influence the results.

**Table 2:** Efficacy of various disinfectants against selective pathogens *in vitro*/*in vitro* with organic matter (“+” = efficacy of all disinfectant components across all studies is 100%; “.” = efficacy of all disinfectant components across all studies is ≥99.99%; “−” = efficacy of all disinfectant components across all studies is <99.99%; “n.a.” = no data available).

	QACs	Aldehydes	Phenol	Hypochlorites	Potassium PMS	Other peroxides	Organic acids	Iodine
*Campylobacter*	+/+	+/+	n.a./+	+/−	n.a./+	n.a./+	n.a./n.a.	+/−
*E. coli*	+/−	+/−	+/−	−/−	−/−	−/+	+/+	−/−
*Enterococcus*	n.a./n.a.	n.a./n.a.	+/n.a.	n.a./n.a.	+/n.a.	+/n.a.	n.a./n.a.	n.a./n.a.
*Salmonella*	+/−	./+	+/−	./−	−/−	+/−	+/−	./n.a.
*Staphylococcus*	+/−	−/−	+/−	+/−	−/−	+/n.a.	n.a./−	−/−

Abbreviations: QACs, quaternary ammonium compounds; PMS, peroxymonosulfate.

**Table 3:** Effectiveness of various disinfectants against selective pathogens on site based on the highest reported effectiveness (indicators before the slash: “+” = highest reported effectiveness is 100%; “.” = highest reported effectiveness is ≥99.99%; “−” = highest reported effectiveness is <99.99%) or on the proportion of papers reporting said level of effectiveness (indicators after the slash: “+” more than half of the studies reported an effectiveness of ≥99.99%; “.” = more than half of the studies reported an effectiveness of ≥90.00%; “−” = more than half of the studies reported an effectiveness of <90.00%).

	QACs	Aldehydes	Phenol	Hypochlorites	Potassium PMS	Other peroxides	Organic acids	Iodine
*Campylobacter*	+/−	+/+	+/−	n.a./n.a.	+/−	−/−	n.a./n.a.	n.a./n.a.
*E. coli*	+/.	+/+	−/.	+/+	n.a./n.a.	+/+	+/+	−/.
*Enterococcus*	+/.	+/.	−/−	n.a./n.a.	−/.	n.a./n.a.	−/−	n.a./n.a.
*Salmonella*	+/.	+/.	−/.	+/.	n.a./n.a.	+/+	+/.	−/.
*Staphylococcus*	−/−	+/+	−/−	n.a./n.a.	n.a./n.a.	+/+	+/+	−/−

Abbreviations: QACs, quaternary ammonium compounds; PMS, peroxymonosulfate.

Based on our numeric quantitative analysis, most disinfectants (aldehyde-based, QAC-based, phenol-based, hypochlorite-based, oxidizing agent–based, organic acid–based compounds) can be recommended whereas potassium peroxymonosulfate and iodine proved generally insufficient for disinfection *in vitro* and in broiler barns.

### Campylobacter

As previously mentioned, 15 papers mentioned cleaning or disinfection measures or both against *Campylobacter*. Of those papers, 11 focused solely on *Campylobacter* whereas the other 4 also investigated additional pathogens. Although there were no notable countries or journals in which papers regarding *Campylobacter* were more frequent than in others, there was a 10-year gap, from 2007 to 2016, in which no relevant paper could be found. Epidemiological data from Liu et al. (2022) has shown an increase in campylobacteriosis in some countries following 2015, which could have rekindled the interest in this pathogen. The average information density was 45.05%, with 1 paper providing all information and 1 no specifics at all. The most neglected factors were the water temperature for cleaning and the time between cleaning and disinfection, which were reported 2 and 3 times, respectively.

From the results of our analysis, shown in more detail in the supplementary data, we can draw several conclusions and point out important risk factors: As seen in most presented studies, disinfection is usually able to reduce *Campylobacter* to varying degrees. Most notable are QACs, to which *Campylobacter* species generally show high susceptibility (Beier et al., 2021), but especially in combination with glutaraldehyde, QACs seem to be able to completely and consistently destroy the pathogen (Abdelal et al., 2016a, 2016b; Battersby et al., 2017; Castañeda-Gulla et al., 2020; Aidaros et al., 2022). The correct concentration of the disinfection agent as shown in Beier et al. (2021) and Aidaros et al. (2022) and the proper amount as shown in Payne et al. (2005) are important for successful disinfection. The kind of water used for cleaning seems to be important, because groundwater in van de Giessen et al. (1996) and geothermal water in Guerin et al. (2007) were risk factors. Although detergents seem somewhat important in reducing the risk (van de Giessen et al., 1996; Gibbens et al., 2001), a detergent itself cannot destroy *Campylobacter* (Battersby et al., 2017; de Castro Burbarelli et al., 2017). Furthermore, findings from Battersby et al. (2017) underline the impact of the application method because *Campylobacter* survived 2 different disinfectants when these were applied by spraying but was completely eradicated when these disinfectants were applied by fogging. No new disinfectants or disinfection methods were tested against *Campylobacter*.

Seven of the 15 papers included usable efficacy or effectiveness data on eradication of *Campylobacter*. According to our search string, Payne et al. (2005) and Aidaros et al. (2022) were the only studies determining the efficacy *in vitro*. In the absence of organic matter (*n* = 1; Aidaros et al., 2022), disinfectants containing QACs, aldehydes, phenol, sodium hypochlorite, or iodine could achieve full disinfection. When organic matter was introduced, QACs, phenol, and aldehydes (*n* = 2; Payne et al., 2005; Aidaros et al., 2022) remained effective at appropriate concentrations. Nascent oxygen and potassium peroxymonosulfate used by Payne et al. (2005) showed full efficacy as well. Sodium hypochlorite showed a small reduction in efficacy to 99.70%, whereas the efficacy of iodine, depending on concentration, was reduced to 61.00% (Aidaros et al., 2022).

The on-site data suggested that QACs (*n* = 3; Abdelal et al., 2016a; Battersby et al., 2017; Castañeda-Gulla et al., 2020) and aldehydes (*n* = 5; van de Giessen et al., 1998; Abdelal et al., 2016a; Battersby et al., 2017; de Castro Burbarelli et al., 2017; Castañeda-Gulla et al., 2020) can achieve full disinfection. However, their success is not guaranteed (*n* = 3; van de Giessen et al., 1998; Battersby et al., 2017; Castañeda-Gulla et al., 2020) and prevalence after disinfection can be as high as 20%. Phenol-containing disinfectants can also be successful (*n* = 1; de Castro Burbarelli et al., 2017) but tend to be insufficient when used without further compounds (*n* = 2; van de Giessen et al., 1998; Abdelal et al., 2016a). According to Battersby et al. (2017), potassium peroxymonosulfate can disinfect properly, but only under certain conditions, those being high concentration and application by fogging, otherwise not. Hydrogen peroxide is ineffective with an effectiveness of less than 60% (*n* = 1; Battersby et al., 2017).

Based on the presented data, we recommend implementing a cleaning protocol involving the use of a detergent as well as fogging a disinfectant with glutaraldehyde and QACs in the correct concentration and an application amount of 1 L/m^2^ if a barn struggles with *Campylobacter* infection of the same serotype, which indicates a possible lack of success during cleaning.

### Escherichia coli

Twenty-six papers involved cleaning or disinfection measures or both against *E. coli*. Of those papers, 8 focused solely on *E. coli* whereas the other 18 also considered further pathogens. There were no notable journals or times in which papers regarding *E. coli* were more frequent. Ten papers, mostly ones with multiple pathogens, were from Egypt. The publication years of those papers were relatively far apart, so no assumption about why they showed heightened interest in *E. coli* research is possible. The average information density was 43.88%, with 1 paper providing all information and 2 giving no specifics. The most neglected factors were the water temperature for cleaning and the time between cleaning and disinfection, which were reported 3 times each.

From the results of our analysis, shown in more detail in the supplementary data, we can draw several conclusions: Formalin could eliminate *E. coli* in the presence of organic matter (Moustafa Gehan et al., 2009; Soliman et al., 2009; Kaoud et al., 2020; Maertens et al., 2020) whereas other disinfectant compounds could not (Sotohy et al., 2021; Aidaros et al., 2022), thus making formalin an appealing choice for on-farm disinfection, where the absence of organic matter cannot be guaranteed. However, as shown in Roedel et al. (2021), some strains had a heightened resistance to formaldehyde, wherefore it is not universally usable. The results for disinfectants with QACs were inconclusive, and Roedel et al. (2021) and Enany et al. (2023) showed that certain strains of *E. coli* are more resistant than others to QACs. Data regarding detergent efficacy or effectiveness is split. Some studies reported a complete eradication of the pathogen when a detergent and the same disinfectant compounds were used (Rathgeber et al., 2009; Ibrahim et al., 2023), whereas others reported minor or no effects on *E. coli* (Luyckx et al., 2015a, 2015b; Abdelal et al., 2016b; Castañeda-Gulla et al., 2020). With the kind of detergent varying, sodium hydroxide was used in multiple studies with similar application methods to varying degrees of effectiveness (Rathgeber et al., 2009; Luyckx et al 2015a, 2015b). The finding that complete disinfection is possible allows the hypothesis that additional factors such as soaking time, application time, temperature, etc. have an important effect, which needs to be researched to maximize effectiveness. Two novel ideas were tested: A combination of sodium chloride and sodium bisulfate showed some effectiveness in a field study (Payne et al., 2019), and zinc oxide nanoparticles were highly effective *in vitro* (Ahmed et al., 2024).

Of the papers available, 18 had usable data regarding disinfection efficacy or effectiveness against *E. coli*. The efficacy of aldehydes and QACs *in vitro* was 100% (*n* = 7; Sander et al., 2002; Moustafa Gehan et al., 2009; Soliman et al., 2009; Sotohy et al., 2021; Aidaros et al., 2022; Abou-Khadra et al., 2024; Ahmed et al., 2024) with 1% being the appropriate concentration. For both compounds, mixed or used alone, Kaoud et al. (2020) reported nearly complete disinfection. Phenolic disinfectants were also described to be effective (*n* = 3; Sander et al., 2002; Sotohy et al., 2021; Aidaros et al., 2022). Hypochlorites were effective in Abd-Elall et al. (2023) but not in Aidaros et al. (2022), which could be due to the difference in concentrations used (5% and 1%, respectively). For peroxide-based disinfectants, Sander et al. (2002) and Moustafa Gehan et al. (2009) described successful disinfection, whereas Abd-Elall et al. (2023) and Ahmed et al. (2024) reported failure, a discrepancy not explicable by differences in concentration. In the study by Moustafa Gehan et al. (2009), organic acids were used additionally, possibly enhancing the efficacy of peroxide compounds. Potassium peroxymonosulfate tended to be unsuccessful (*n* = 4; Moustafa Gehan et al., 2009; Kaoud et al., 2020; Sotohy et al., 2021; Abd-Elall et al., 2023) and rarely showed success in the eradication of *E. coli* (*n* = 2; Sotohy et al., 2021; Ahmed et al., 2024). Iodine-based disinfectants showed 100% efficacy in Soliman et al. (2009), nearly complete efficacy in Kaoud et al. (2020), and less than 70% efficacy depending on concentration in Aidaros et al. (2022). Concentration seems to be important, and a concentration of 7.50% iodine is recommended.

The results of QACs for disinfection of *E. coli* were negatively impacted by organic matter (*n* = 3; Moustafa Gehan et al., 2009; Kaoud et al., 2020; Aidaros et al., 2022), but successful disinfection was also reported (*n* = 3; Moustafa Gehan et al., 2009; Soliman et al., 2009; Aidaros et al., 2022). Aldehydes tended to remain effective at the appropriate concentration in some studies (*n* = 3; Moustafa Gehan et al., 2009; Soliman et al., 2009; Aidaros et al., 2022) and showed slightly reduced performance in Kaoud et al. (2020). Depending on the concentration, phenolic compounds in combination with other compounds showed full disinfection efficacy in the presence of organic matter (Aidaros et al., 2022). The hypochlorite compounds tested in Aidaros et al. (2022) showed 95% efficacy. Moustafa Gehan et al. (2009) and Kaoud et al. (2020) reported insufficient efficacy of potassium peroxymonosulfate against *E. coli*. Hydrogen peroxide, alone and in combination with organic acids, prevented the growth of *E. coli* in Moustafa Gehan et al. (2009). Although iodine could achieve full disinfection *in vitro* without and with the addition of organic matter (*n* = 1; Soliman et al., 2009), it usually showed low efficacy (*n* = 3; Abdelal et al., 2016b; Kaoud et al., 2020; Aidaros et al., 2022).

In on-site field studies, QACs showed both success (*n* = 3; Rathgeber et al., 2009; Luyckx et al., 2015a; Ibrahim et al., 2023) and failure (*n* = 3; Luyckx et al., 2015b; Abdelal et al., 2016a; Castañeda-Gulla et al., 2020) in eliminating *E. coli*. In Luyckx et al. (2015b), disinfection effectiveness was strongly location based, ranging from 100% to 47.00%. The combined application of QACs and aldehydes also showed both success (*n* = 4; Rathgeber et al., 2009; Luyckx et al., 2015a; Abdelal et al., 2016b; Ibrahim et al., 2023) and failure (*n* = 3; Luyckx et al., 2015b; Abdelal et al., 2016a; Castañeda-Gulla et al., 2020). Phenol-containing disinfectants failed to achieve full disinfection (*n* = 2; Abdelal et al., 2016a, 2016b). Despite the mixed results *in vitro*, Rathgeber et al. (2009) and Abdelal et al. (2016b) showed that disinfection based solely on sodium hypochlorite or in combination with other compounds can be effective on site. Peroxides (*n* = 2; Rathgeber et al., 2009; Badr and Yoseif, 2014) and organic acids (*n* = 2; Kašková et al., 2006; Badr and Yoseif, 2014) were effective in field studies. Iodine showed limited effectiveness in Abdelal et al. (2016b) and no effect in Rathgeber et al. (2009).

Therefore, if a barn faces repeated *E. coli* infections, we recommend a test on QAC or formalin susceptibility to prevent resistance development due to ineffective or low doses of active ingredients and to increase broiler health and reduce antibiotic use as a result of successful disinfection.

### Salmonella

Roughly 50% (*n* = 35) of all papers involved cleaning or disinfection measures or both against *Salmonella*. Of those papers, 22 focused solely on *Salmonella* whereas 13 also investigated additional pathogens. There were no notable journals or times in which papers regarding *Salmonella* were especially frequent. Five papers, mainly ones dealing with multiple pathogens, came from Egypt and 4 from France. The publication years were relatively far apart, allowing no assumption about the reason for heightened interest in research on *Salmonella*. The average information density was 41.50%, with 2 papers providing all information and 5 giving no specifics. The most neglected factors were the water temperature for cleaning and the time between cleaning and disinfection, which were reported 4 times each.

From the results of our analysis, shown in more detail in the supplementary data, we drew several conclusions and identified several important factors: The use of a detergent in cleaning protocols was important according to Cardinale et al. (2004) and Badr and Yoseif (2014), and disinfectants lost effectiveness in the presence of organic matter in Stringfellow et al. (2009). Three disinfectants had the highest efficacy or effectiveness across nearly all studies. These were aldehydes (Gradel et al., 2005; Marin et al., 2011; Abdelal et al., 2016a, 2016b; Aksoy et al., 2020; Castañeda-Gulla et al., 2020; Drauch et al., 2020; Newton et al., 2020; Sotohy et al., 2021; Abd-Elall et al., 2023; Kranjc et al., 2024; Sevilla-Navarro et al., 2024), QACs (Sander et al., 2002; Moustafa Gehan et al., 2009; Stringfellow et al., 2009; Castañeda-Gulla et al., 2020; Drauch et al., 2020; Sotohy et al., 2021; Sevilla-Navarro et al., 2024), and sodium hypochlorite (Kaoud and Yosseif, 2013; Abdelal et al., 2016a; Kloska et al., 2017; Bassani et al., 2021; Abd-Elall et al., 2023). Four papers presented new disinfection options: Bacteriophages, especially with common disinfectants afterwards (Sevilla-Navarro et al., 2024), slightly acidic electrolyzed water (Shi et al., 2020), added silver nanoparticles (Kaoud and Yosseif, 2013), and *Thymus vulgaris* essential oil (Sperandio et al., 2023) were very effective in removing *Salmonella*, whereas the monoterpene thymol proved ineffective (Sperandio et al., 2023).

The level of efficacy or effectiveness of disinfectants against *Salmonella* was mentioned in 18 papers. *In vitro*, all disinfectants containing QACs were successful (*n* = 5; Sander et al., 2002; Moustafa Gehan et al., 2009; Stringfellow et al., 2009; Aksoy at al., 2020; Sotohy et al., 2021). Aldehydes succeeded most of the time (*n* = 4; Moustafa Gehan et al., 2009; Aksoy at al., 2020; Sotohy et al., 2021; Abd-Elall et al., 2023) and showed almost complete efficacy in Kaoud and Yosseif (2013). Phenolic compounds (*n* = 3; Sander et al., 2002; Stringfellow et al., 2009; Sotohy et al., 2021), organic acids (Moustafa Gehan et al., 2009), peroxides (*n* = 4; Sander et al., 2002; Moustafa Gehan et al., 2009; Aksoy at al., 2020; Abd-Elall et al., 2023), and sodium hypochlorite (Abd-Elall et al., 2023) were effective *in vitro*. Iodine showed 100% efficacy *in vitro* in Aksoy et al. (2020) and failed to achieve 100% efficacy in Kaoud and Yosseif (2013). Potassium peroxymonosulfate succeeded in Sotohy et al. (2021) and failed otherwise (*n* = 2; Moustafa Gehan et al., 2009; Abd-Elall et al., 2023).

If organic material was introduced in the *in vitro* testing, some studies reported 100% efficacy of QAC-based disinfectants (*n* = 3; Moustafa Gehan et al., 2009; Stringfellow et al., 2009; Drauch et al., 2020) whereas others reported reduced efficacy (*n* = 4; Payne et al., 2005; Moustafa Gehan et al., 2009; Stringfellow et al., 2009; Bassani et al., 2021). According to Moustafa Gehan et al. (2009), aldehyde-containing disinfectants remain effective against *Salmonella* even in the presence of organic matter. Phenol showed 100% efficacy in Stringfellow et al. (2009) and failed otherwise (*n* = 2; Payne et al., 2005; Drauch et al., 2020). Bassani et al. (2021) reported successful elimination of *Salmonella* by using sodium hypochlorite, and Kaoud and Yosseif (2013) reported almost complete efficacy of a hypochlorite mixture in the presence of organic matter. Potassium peroxymonosulfate showed little to no effect if organic matter was introduced (*n* = 3; Payne et al., 2005; Moustafa Gehan et al., 2009; Drauch et al., 2020), only succeeding once in Drauch et al. (2020). Other peroxide-based disinfectants failed in some tests (*n* = 2; Payne et al., 2005; Drauch et al., 2020) and succeeded in others (*n* = 2; Moustafa Gehan et al., 2009; Drauch et al., 2020). Organic acids remained effective in the presence of organic matter (*n* = 2; Moustafa Gehan et al., 2009; Drauch et al., 2020) but failed when high concentrations of organic matter were present (Drauch et al., 2020).

On-site data showed that QACs can be successful in removing *Salmonella* under certain circumstances (Sevilla-Navarro et al., 2024), but they usually fail (*n* = 3; Luyckx et al., 2015b; Abdelal et al., 2016a; Castañeda-Gulla at al., 2020) with effectiveness reduced to as little as 81.81% (Luyckx et al., 2015b). Aldehyde-containing disinfectants showed 100% effectiveness in some studies (*n* = 4; Abdelal et al., 2016b; Kloska et al., 2017; Newton et al., 2020; Sevilla-Navarro et al., 2024,) and low effectiveness in others (*n* = 7; Lahellec et al., 1986; Marin et al., 2011; Luyckx et al., 2015b; Abdelal et al., 2016a, 2016b; Kloska et al., 2017; Castañeda-Gulla et al., 2020). Phenol was unsuccessful when used on site (*n* = 3; Lahellec et al., 1986; Abdelal et al., 2016a, 2016b). In Abdelal et al. (2016b), a hypochlorite-based disinfectant was successful at high concentration and not fully reliable at lower concentration. In Sevilla-Navarro et al. (2024), hydrogen peroxide was successful in removing remaining *Salmonella* after prior experimental treatment. Peracetic acid alone was unable to fully disinfect and was very effective in combination with sodium hydroxide (Kloska et al., 2017). Abdelal et al. (2016b) could not achieve full disinfection with the usage of iodine-based disinfectants.

Based on the presented data, we recommend implementing a cleaning protocol involving the use of a detergent and a disinfectant with aldehydes or QACs or both and finishing with sodium hypochlorite in the correct concentration and amount if a barn repeatedly shows *Salmonella* infection of the same serotype, indicating a possible lack of success during cleaning.

### Staphylococcus

Sixteen papers mentioned cleaning or disinfection measures or both against *Staphylococcus*. Of those papers, 1 focused solely on *Staphylococcus* whereas the other 15 also investigated additional pathogens. More than half of the papers were affiliated to Egypt. There was no specific data for *Staphylococcus* infections or an increase in broiler production to allow an interpretation of the relatively high amount of research in Egypt. The average information density was 44.90%, with no paper providing all the information. The majority of neglected factors were the water temperature for cleaning and the time between cleaning and disinfection, which were not reported in any paper. Most studies conducted *in vitro* tests or tested a novel idea, whereas none dealt with potential risk factors. Two novel ideas were presented: Added silver- or copper nanocomposites (Elsayed et al., 2020) and the use of slightly acidic electrolyzed water (Shi et al., 2020) were successful in eliminating the pathogen.

From the results of our analysis, shown in more detail in the supplementary data, no specific recommendation or conclusion can be made. Although most disinfectants were effective to varying degrees against *Staphylococcus* species, there was a lot of conflicting information on how effective the active ingredients were. Disinfectants containing potassium peroxymonosulfate, for example, were evaluated as very effective by Moustafa Gehan et al. (2009) and Sotohy et al. (2021). However, Elsayed et al. (2020) and Abd-Elall et al. (2023) reported a lower effectiveness. Whereas QACs seemed to be effective in *in vitro* tests done by Aidaros et al. (2022) and Abou-Khadra et al. (2024), they failed to significantly reduce *Staphylococcus* in Castañeda-Gulla et al. (2020), who operated under field conditions on site. Because other pathogens in the latter study were reduced and some even eliminated, the lack of effectiveness was specific, and biofilms or the *Staphylococcus* genotype and previous exposure to the compounds might have been relevant confounding factors. Therefore, without further data and research, QACs cannot be recommended as effective disinfectants for broiler farms with repeated *Staphylococcus* infections.

Overall, 13 studies provided usable data regarding the efficacy or effectiveness of commercially relevant disinfectant compounds for eradication of *Staphylococcus*. QACs succeeded in all *in vitro* tests with no organic matter present (*n* = 7; Sander et al., 2002; Moustafa Gehan et al., 2009; Soliman et al., 2009; Stringfellow et al., 2009; Sotohy et al., 2021; Aidaros et al., 2022; Abou-Khadra et al., 2023). Whereas aldehydes achieved full disinfection most of the time (*n* = 7; Sander et al., 2002; Moustafa Gehan et al., 2009; Soliman et al., 2009; Stringfellow et al., 2009; Sotohy et al., 2021; Aidaros et al., 2022; Abou-Khadra et al., 2023), their efficacy was 70% in 1 study (Abd-Elall et al., 2023). Disinfectants containing phenol (*n* = 4; Sander et al., 2002; Stringfellow et al., 2009; Sotohy et al., 2021; Aidaros et al., 2022), hydrogen peroxide (*n* = 3; Sander et al., 2002; Moustafa Gehan et al., 2009; Abd-Elall et al., 2023), organic acids (Moustafa Gehan et al., 2009), or sodium hypochlorite (Abd-Elall et al., 2023) also showed 100% efficacy. Iodine was effective in 1 study (Soliman et al., 2009) and failed in another (Aidaros et al., 2022). The disinfectant based on potassium peroxymonosulfate tested by Abd-Elall et al. (2023) failed with 91.4% efficacy, whereas those tested by Moustafa Gehan et al. (2009) and Sotohy et al. (2021) achieved full disinfection.

Despite several QAC- and aldehyde-based disinfectants remaining effective against *Staphylococcus* in the presence of organic matter (*n* = 4; Moustafa Gehan et al., 2009; Soliman et al., 2009; Stringfellow et al., 2009; Aidaros et al., 2022), some showed compromised efficacy (*n* = 3; Stringfellow et al., 2009; Elsayed et al., 2020; Aidaros et al., 2022) reduced to as little as 86.40% (Aidaros et al., 2022). Phenol-based disinfectants (*n* = 3; Stringfellow et al., 2009; Elsayed et al., 2020; Aidaros et al., 2022), sodium hypochlorite (*n* = 2; Elsayed et al., 2020; Aidaros et al., 2022), and potassium peroxymonosulfate (*n* = 2; Moustafa Gehan et al., 2009; Elsayed et al., 2020) also lost their efficacy in the presence of organic matter. According to Moustafa Gehan et al. (2009), disinfectants based on hydrogen peroxide and those based on organic acids remain effective. Elsayed et al. (2020) reported very high efficacy of organic acids in the presence of organic matter if dosed high enough, otherwise efficacy was as low as 72.40%. Results with iodine-based disinfectants were mixed with 1 success (Soliman et al., 2009) and 1 failure in the presence of organic matter (Aidaros et al., 2022).

On-site applications of QAC-based disinfectants did not achieve success across the available data (*n* = 2; Abdelal et al., 2016a; Castañeda-Gulla et al., 2020), with effectiveness as low as 96.67% (Abdelal et al., 2016a). Aldehydes mostly showed low levels of effectiveness (*n* = 3; Abdelal et al., 2016a, 2016b; Castañeda-Gulla et al., 2020) and 100% effectiveness in 1 study (Abdelal et al., 2016b). Phenol-containing disinfectants failed to achieve acceptable levels of effectiveness (*n* = 2; Abdelal et al., 2016a, 2016b), going as low as 80.00%. The only effective disinfectant on site was a hydrogen peroxide–organic acid mixture described by Badr and Yoseif (2014).

Owing to the lack of data, no specific recommendation for broiler farms facing repeated *Staphylococcus* infections can be made from the reported information.

### Others

In total, 27 papers mentioned cleaning or disinfection measures or both against pathogens other than *E. coli, Campylobacter, Staphylococcus,* and *Salmonella*. Each of the “other” pathogens was mentioned in fewer than 10 papers. Of those 27 papers, 10 focused solely on 1 pathogen and 17 investigated multiple pathogens. Research in 8 papers was affiliated to Egypt. The average information density was 55.00%, with 2 papers providing all the information and 1 giving no specifics. The most neglected factor was the water temperature for cleaning, which was reported in 6 papers. Most papers (*n* = 20) included field tests conducted on site instead of *in vitro* tests.

From the results of our analysis, shown in more detail in the supplementary data, we cannot make specific recommendations or draw conclusions owing to the small numbers of studies on any “other” pathogen. Nevertheless, the results show that different pathogens react differently to cleaning and disinfection measures, highlighting the need for more selective disinfection efforts if a certain kind of pathogen causes repeated issues in the same barn.

Despite *Enterococcus* being mentioned in an insufficient number of papers (<10) to give this genus a separate heading, all 9 sources presented usable data. Both Sander et al. (2002) and Luyckx et al. (2017) performed *in vitro* tests that showed the efficacy of peroxide-based, phenol-based, and organic acid–based disinfectants. No data regarding the presence of organic matter was available. QACs-containing disinfectants achieved complete (Luyckx et al., 2015a) or nearly complete disinfection (Mateus-Vargas et al., 2022) and proved to be ineffective against *Enterococcus* on multiple occasions (*n* = 2; Garcia-Migura et al., 2007; Luyckx et al., 2015b). Similarly, aldehydes achieved complete (*n* = 2; Nilsson et al., 2013; Luyckx et al., 2015a) or nearly complete disinfection (Mateus-Vargas et al., 2022) or underperformed considerably (*n* = 3; Nilsson et al., 2013; Luyckx et al., 2015b; Tessin et al., 2024). Phenolic compounds (Garcia-Migura et al., 2007), potassium peroxymonosulfate (Ward et al., 2006), and organic acids (*n* = 2; Garcia-Migura et al., 2007; Tessin et al., 2024) were not effective against *Enterococcus*.

Whereas QACs and aldehydes could achieve full disinfection *in vitro*, the on-site results were mixed, possibly indicating differences in sampling methods, application methods, or other factors. Thus, more research is necessary to develop a reliable cleaning and disinfection protocol against *Enterococcus*.

### Limitations

A limitation of the present review is the absence of a formal risk-of-bias or study-quality assessment, such as ARRIVE (Percie du Sert et al., 2020), STROBE (von Elm et al., 2007), or GRADE (Guyatt et al., 2008). The majority of the reviewed studies did not provide sufficient methodological detail to allow for such evaluation. Critical parameters such as water temperature, detergent use, exposure time, and surface type were often incompletely reported or entirely missing. As a result, the calculated “effectiveness” scores may partly reflect differences in study design and reporting quality rather than the true antimicrobial activity of the disinfectants, highlighted by Luyckx et al. (2015b), where sampling location, especially the more uncommon ones, meant the difference between full effectiveness and failure of the disinfectant. Furthermore, as stated in the Materials and Methods section of our paper, greatly differing sampling techniques and measurements of success were used. Nevertheless, even excluding all studies with incomplete information regardless of sampling or measurement type substantially reduced the available dataset and prevented a meaningful synthesis of the literature.

By including all relevant publications, the review provides a comprehensive overview of the current evidence base while transparently acknowledging existing data gaps. This situation highlights the urgent need for more standardized and detailed reporting of disinfection trials in broiler production, which would allow future reviews to apply formalized quality-assessment frameworks and strengthen the comparability of results across studies. The lack of detailed reporting of key contextual parameters such as organic load, surface type, water hardness, temperature, contact time, and even proper disinfectant documentation regarding specific compounds and applied concentration represents a major limitation of the available literature. In our dataset, such information was missing in nearly 80% of the publications, preventing a meaningful statistical analysis or modeling of their influence on disinfection efficacy or effectiveness—again, highlighting the urgent need for standardized reporting of these critical parameters.

### Summary

The recommendations based on the available data are summarized in Table 2, Table 3. Furthermore, the correct concentration (e.g., Sotohy et al., 2021; Aidaros et al., 2022), the correct application amount (e.g., Payne et al., 2005; Battersby et al., 2017), and the use of detergent (e.g., van de Giessen et al., 1996) are important for successful elimination of pathogens. Although traditional disinfectants are still mostly very effective, novel ideas (e.g., Sperandio et al., 2023; Ahmed et al., 2024; Sevilla-Navarro et al., 2024) offer new and successful additions in the face of increasing disinfection resistance (Enany et al., 2023).

## Conclusion

Three major conclusions can be drawn from the presented data: First, although most disinfectants are generally effective, some bacteria such as *Campylobacter* and *Salmonella* seemingly react differently to certain disinfectants. For example, sodium hydroxide has less impact against *Campylobacter* and phenol is less effective against *Salmonella*, despite both disinfectants being highly effective against the other pathogens. New technologies, especially nanoparticles, seem to be very promising as an additive if not even a replacement for current disinfectants. Nevertheless, although most *in vitro* tests have shown a general efficacy of nearly all disinfectants, the on-farm applications have rarely resulted in complete eradication of the pathogens. These data show that the effectiveness of the disinfectants is lost there. One reason could be the remaining organic matter, because the importance of using detergents was highlighted in several studies.

Second, although it should be obvious that parameters such as detergent use, state of the barn prior to disinfection, time for the disinfectant to act, and the type of disinfectant have a major impact on the success of disinfection, a lot of parameters are rarely reported in the papers. This results in a lack of knowledge and prevents identifying potential risk factors, which could be easily resolved.

Third, besides having no fixed application rate, the variety of disinfectant components in commercial products makes disinfection, as well as recommendations, difficult. Although the component might generally be effective against the pathogen, the used concentration might be insufficient. As seen in some *E. coli* cases, some strains are starting to become resistant to some compounds. Inadequate dosage could increase the selection of those pathogens and render the component completely useless, as is the problem with numerous antibiotics.

We highly recommend performing a test of susceptibility to the used disinfectant to make sure that the disinfectant can eliminate the pathogen, especially if a barn faces repeated pathogen infections of the same serotype, indicating a possible lack of success during cleaning. Furthermore, because it has been shown repeatedly that some disinfectants fail in the presence of organic matter, the cleaning protocol should be evaluated as well.

## Declaration of generative AI and AI-assisted technologies in the writing process

During the preparation of this work, the authors used DeepL Write (version from 14.01.2025) and ChatGPT (GPT-4-Turbo) to improve language and readability, while not using them to generate text. After using these tools or services, the authors have reviewed and edited the content as needed, and they take full responsibility for the content of the publication.

## Ethical statement

The work presented in this review did not include research on live animals.

## CRediT authorship contribution statement

**Adrian Drögmöller:** Writing – review & editing, Writing – original draft, Methodology, Investigation, Formal analysis, Data curation. **Helen Louton:** Writing – review & editing, Visualization, Supervision, Methodology, Conceptualization.

## Disclosures

The authors declare no conflict of interest. The funder of the project had no influence on the content and design of the manuscript. We confirm that the manuscript has been read and approved by all named authors and that there are no other persons who satisfied the criteria for authorship but are not listed. We further confirm that the order of authors listed in the manuscript has been approved by all the authors.

## Appendix Supplementary materials

Image, application 1
