# Supplementary material for: Clean sweep or pathogen paradise? A critical review of cleaning and disinfection practices in broiler barns
Source: Poult Sci. 2025 Dec 11;105(2):106232. doi: 10.1016/j.psj.2025.106232 (PMC12767818; doi:10.1016/j.psj.2025.106232)
Supplement: Supplementary file 1 [file mmc1.docx]

| Pathogen | Study | Cleaning | Disinfection | Results |
| --- | --- | --- | --- | --- |
| *Campylobacter* | Abdelal et al. 2016 | - Farm B: Biosafe 3% | - Farm A: phenol 5%, spayed - Farm B: Virocide 0.50% (Glutaraldehyde, Quaternary ammonium compounds, other), sprayed | - 20% of relevant samples from Farm A were positive for *Campylobacter*, while none in Farm B. |
| *Campylobacter* | Aidaros et al. 2022 | (*in vitro* test) | - Prophyl 2000® 0.40%, 2%, 4% (Glutaraldehyde, Quaternary ammonium compounds, Phenol) - G7® 0.25% 0.50%,1% (Glutaraldehyde, Quaternary ammonium compounds) - Pron-Tech® 0.10%, 0.20%, 0.05% (Quaternary ammonium compounds, urea) - Alkadox® 0.50%, 1%, 1.5% (Sodium hypochlorite, sodium carbonate) - Biodine® 0.50%, 1%, 1.5% (Iodine, other) | - Prophyl 2000®, G7®, as well as Pron-Tech® can eliminate all Campylobacter within 90 minutes or less, even in the presence of organic material within their recommended concentration. - Alkadox® and Biodine® needed to surpass their recommended concentration to achieve total disinfection of *Campylobacter* in the presence of organic material. |
| *Campylobacter* | Battersby et al. 2017 | - Sodium hydroxide 1%, 5%, foam | - Potassium peroxymonosulfate, Sulfamic acid, Sodium chloride 1%, sprayed - Potassium peroxymonosulfate, Sulfamic acid, Sodium chloride 5%, fogged - Hydrogen peroxide, d-Limonene 1%, sprayed - Glutaraldehyde, Quaternary ammonium compounds 0.50%, sprayed - Glutaraldehyde, Quaternary ammonium compounds 0.30%, fogged | - Glutaraldehyde with Quaternary ammonium compounds (0.30%, fogged), as well as the combination of potassium peroxymonosulfate, sulfamic acid, and sodium chloride (5%, fogged), both showed the best results, with only 11.11% of the samples remaining positive. - The other disinfectants, as well as the detergents alone, were inadequate in eliminating Campylobacter, with over 33.33% of samples remaining positive after treatment. - Note: The fogged disinfection methods had the highest application amount with 1l/m^2^ instead of 0.30l or less for the rest. Therefore, the amount of disinfectant varies, and no conclusion can be drawn regarding the effectiveness of the application method. |
| *Campylobacter* | Beier et al. 2021 | (*in vitro* test) | - Varying, including: - Formaldehyde - Quaternary ammonium compounds | - The minimum inhibitory concentration 90 for Formaldehyde is 32 μg/mL. - Most Quaternary ammonium compounds are highly effective against *Campylobacter* with a minimum inhibitory concentration90 of 1 or 4 μg/mL, depending on the type. - The exception is Benzyldimethylhexadecylammonium chloride, which requires 16 μg/mL. - Note: Other disinfections, which are not typically used by cleaning procedures in the poultry industry, were looked at too. |
| *Campylobacter* | Berndtson et al. 1996 | - Mostly mechanically, followed by high-pressure with cold water and without detergent | - Several Days after cleaning, fogging with aldehydes or other disinfectants | - In 64 cases of *Campylobacter* *spp.* detection only 25% showed reinfection. |
| *Campylobacter* | Castañeda-Gulla et al. 2020 | - Blown, high-pressure, Biostrip 3000 | - Glutasan QCT (Glutaraldehyde, Quaternary ammonium compounds), high pressure | - Depending on the cycle and location, between 0% and 30% were positive after disinfection. |
| *Campylobacter* | de Castro Burbarelli et al. 2017 | - A: Neutral detergent - B: Sweeping, high pressure, alkaline and acid detergents (4%), ≈0.40l/m^2^ | - B: After partially drying, Glutaraldehyde (250g/l), formaldehyde (185g/l), 0.50%, sprayed, ≈0.40l/m^2^, para-chloro-meta-cresol (Phenol, 210g/l), 4%, ≈0.40l/m^2^ | - Cleaning and disinfection following B significantly decreased (p< 0.05) the amount of positive samples (0%) in comparison to A (30%). - Note: No *Campylobacter* could be found after inoculation of the chicks in the cycle prior, which could have impacted the results. |
| *Campylobacter* | Evans and Sayers 2000 | n.a. | n.a. | - The method of dust removal has no impact on infection time. - Waiting more than 24 hours after cleaning is associated with faster infection time (p<0.2). - Having the farm cleaned by a contractor shows faster infection in comparison to cleaning by farm staff (p<0.2). - Note: The sample size for farms cleaned by farm staff was small. |
| *Campylobacter* | Gibbens et al. 2001 | - varying | - varying | - Blowing as a dust removal practice is shown to be more effective than other methods for dust removal (p<0.2). - Using the recommended concentration of detergent is better than a higher or lower concentration (p<0.2). - Waiting till the barn is dried before disinfection and having six or more hours in between cleaning and disinfection proves effective (p<0.2, p<0.2). - The use of a second disinfectant is associated with a higher risk of faster infection (p<0.2). - Note: The sample size for the use of a second disinfectant is relatively small. - Note: The results were chosen from multiple kinds of tests (Fisher’s exact test/Wilcoxon test). |
| *Campylobacter* | Guerin et al. 2007 | - Geothermal water (hot) | n.a. | - The use of geothermal hot water for cleaning is found to be a risk factor for *Campylobacter* colonization (p<0.05). |
| *Campylobacter* | Mageto et al. 2018 | n.a. | n.a. | - Cleaning and disinfection reduce the risk of *Campylobacter* infection (p < 0.05, OR 0.11). |
| *Campylobacter* | Payne et al. 2005 | n.a. | - Phenol - Potassium-peroxymonosulfate - Nascent oxygen - Quaternary ammonia - Same concentration different application amount: (≈0.108l /m^2^; ≈1.345l/m^2^) | - The low application amount was unsuccessful in eliminating *Campylobacter*, while the higher application amount resulted in no detection of it. |
| *Campylobacter* | Ursinitsch et al. 2005 | n.a. | n.a. | - Wet disinfection only results in 57.2% of flocks being positive instead of 82.1% (OR:3.4) |
| *Campylobacter* | van de Giessen et al. 1996 | n.a. | n.a. | - The use of an unspecified detergent reduces the risk of infection (p< 0.05). - The use of groundwater instead of tap water for cleaning is considered a risk factor (p<0.1) |
| *Campylobacter* | van de Giessen et al. 1998 | n.a. | - Farm A: 20% formalin - Farm B: halamid | - The Formalin usage has shown a reduction from 66% positive flocks to 22% on farm A. - The Halamid usage has shown a reduction from 100% to 42% on farm B. - Note: Other hygiene measures were introduced, too. |
| *Coli* | Abdelal et al. 2016a | n.a. | - Virudox-G (n.a.) - Glutarol (Glutaraldehyde) - Alkadox (Sodium hypochlorite, sodium carbonate) - Swift (n.a.) - Iodoline (Iodine) - Phenodex (Phenol, n.a.) - All et 0.50%, 1%. | - At 0.50% Virudox-G, Glutarol, and Alkadox resulted in a complete reduction, while the rest achieved an effectiveness of 99.8% to 99.9%. - At 1% Iodoline and Phenodex, only achieved an effectiveness of 99.9%, while the rest eliminated the pathogen. |
| *Coli* | Abdelal et al. 2016b | - Farm B: Biosafe 3% | - Farm A: phenol 5%, spayed - Farm B: Virocide 0.50% (Glutaraldehyde, Quaternary ammonium compounds), sprayed | - 6.66% of relevant samples from farm A were positive for *E. coli*, while 2.22% from farm B were. |
| *Coli* | Abd-Elall et al. 2023 | (*in vitro* test) | - Hydrogen peroxide - Sodium hypochlorite - Virkon S (Potassium peroxymonosulfate) - Glutaraldehyde - Copper sulphate - All at 1%, 2%, 5%. | - Only sodium hypochlorite at 2% and 5% was able to eliminate *E. coli*. |
| *Coli* | Abou-Khadra et al. 2024 | (*in vitro* test) | - TH4 ® 2%, 1%, 0.5%, 0.25% (Glutaraldehyde, Quaternary ammonium compounds, other) | - *E. coli* is susceptible to TH4 ®. |
| *Coli* | Ahmed et al. 2024 | (*in vitro* test) | - TH4 ® 1%, 0.5% (Glutaraldehyde, Quaternary ammonium compounds, other) - Virkon S 1%, 0.50% (Potassium peroxymonosulfate) - Hydrogen peroxide 5%, 3% - Zinc oxide nanoparticles 0.04 mg/ml, 0.02 mg/ml - Hydrogen peroxide, zinc oxide nanoparticles composite 0.04 mg/ml, 0.02 mg/ml | - All disinfectants were able to significantly reduce the amount of *E. Coli* (p<0.05). - Except for the hydrogen peroxide, all disinfectants were able to eliminate *E. Coli* after 120min at their respective higher concentration completely. |
| *Coli* | Aidaros et al. 2022 | (*in vitro* test) | - Prophyl 2000® 0.40%, 2%, 4% (Glutaraldehyde, Quaternary ammonium compounds, Phenol) - G7® 0.25% 0.50%,1% (Glutaraldehyde, Quaternary ammonium compounds) - Pron-Tech® 0.10%, 0.20%, 0.50% (Quaternary ammonium compounds, urea) - Alkadox® 0.50%, 1%, 1.5% (Sodium hypochlorite, sodium carbonate) - Biodine® 0.50%, 1%, 1.5% (Iodine, other) | - Only Prophyl 2000® can eliminate all *Campylobacter* within 90 minutes or less, even in the presence of organic material within their recommended concentration. - G7® and Pron-Tech® needed to surpass their recommended concentration to achieve total disinfection of *E.Coli* in the presence of organic material. |
| *Coli* | Castañeda-Gulla et al. 2020 | - Blown, high-pressure, Biostrip 3000 | - Glutasan QCT (Glutaraldehyde and Quaternary ammonium compounds), high-pressure | - *E. Coli* was not significantly reduced (p>0.05), except for the third cycle, where it was not detectable in most areas, crevices, and the annex, being the exception. |
| *Coli* | Course et al. 2021 | n.a. | n.a. | - Disinfection reduces risk of infection (or 0.40, p<0.05). |
| *Coli* | Enany et al. 2023 | (gene test) | (gene test) | - Some *E. Coli* show genetic variations associated with an increased resistance to quaternary ammonium compounds. |
| *Coli* | Gradel et al. 2005 | (*in vitro* test) | - Formaldehyde - Bio Komplet Plus (Glutaraldehyde, Quaternary ammonium compounds) - Virkon S (Potassium-peroxymonosulfate) - Farm Fluid S (phenol) - FAM 30 (Iodophor) | - Farm Fluid S and Formaldehyde don’t significantly impact *E.Coli* (p>0.05). |
| *Coli* | Ibrahim et al. 2023 | - high pressure, Bioshield 3000 | - Bioshield 1% (Glutaraldehyde, quaternary ammonium compound) - Bioshield P 1% (Glutaraldehyde, quaternary ammonium compound) - Bi-OO-Cyst 0.625% (other) | - Following disinfection, no coliform bacteria can be found on the Floor or vents |
| *Coli* | Kašková et al. 2006 | - n.a. | - Peracetic acid 0.40 % | - The peracetic acid was able to eliminate all coliform. |
| *Coli* | Kaoud et al. 2020 | - n.a. | - Verkon- S® 0.83% (Potassium peroxymonosulfate) - Aldekol des- Gda® 0.4% (Glutaraldehyde, quaternary ammonium compound) - Biosentry® 904™ (Quaternary ammonium compound) - TH4® 1% (Glutaraldehyde, quaternary ammonium compound) - Formalin 2.5% - Iodophore (Iodine) 1%; - Phenique (other) 3% | - Except for Iodophore and Phenique, all disinfectants were able to eliminate *E. Coli* in the absence of organic matter. - Only the performance of TH4® and Formalin didn´t seem to be negatively affected by the presence of organic matter. |
| *Coli* | Luyckx et al. 2015b | - high pressure, soap - KenoTM San 1% (Sodium hydroxide) - Intra Power Foam 3% (Sodium hydroxide) - Sodium Hydroxide 50% 1% (Sodium hydroxide) - Ino Net 3% (Sodium hydroxide + potassium hydroxide) - Protocol 1: Overnight soaking and cleaning with warm water - Protocol 2: Overnight soaking and cleaning with cold water - Protocol 3: Cleaning with warm water - Protocol 4: Cleaning with cold water | - Cid 20 2% (Glutaraldehyde, Quaternary ammonium compounds, alcohols), fogging - Desbest 700 1% (Glutaraldehyde, Quaternary ammonium compounds, alcohols), spraying - ViroCid 1.8% (Glutaraldehyde, Quaternary ammonium compounds, alcohols), spraying - Cid 20 2% (Glutaraldehyde, Quaternary ammonium compounds, alcohols), spraying - Hyprelva SL 2% (Glutaraldehyde, Quaternary ammonium compounds, alcohols), fogging | - No difference between the protocols was found. *E. Coli* was reduced to 6-9% positive samples following disinfection. |
| *Coli* | Luyckx et al. 2015a | - Varying see Luyckx et al. 2015b | - Cid 20 2% (Glutaraldehyde, Quaternary ammonium compounds, alcohols), fogging - Desbest 700 1% (Glutaraldehyde, Quaternary ammonium compounds, alcohols), spraying | - After disinfection, only 1-4% of samples remain *E. Coli* positive. |
| *Coli* | Maertens et al. 2020 | n.a. | - Quaternary ammonium compound - Glutaraldehyde - Formaldehyde - D50® (Hydrogen peroxide, other) | - All disinfectants are effective. - Even throughout repeated use over a year, *E. Coli* Isolates show no increased resistance in that timeframe. |
| *Coli* | Mo et al. 2016 | n.a. | n.a. | - Disinfecting after every cycle significantly (p<0,05) reduces the risk of *E. Coli* infection. |
| *Coli* | Moustafa Gehan et al. 2009 | (*in vitro* test) | - Perasan® 1% (Peracetic acid, H2O2, other) - H2O2® 3% (H2O2, other) - Aldekol® 1% (Glutaraldehyde, Quaternary ammonium compounds, Formalin) - Quatovet® 1% - (Quaternary ammonium compounds) - Virkon S® 1% (Potassium peroxymonosulfate) | - Except for Virkon S® 1% all disinfectants were effective in the absence of organic matter. - Virkon S® 1% and Quatovet® 1% were the only ineffective disinfectants in the presence of organic matter. |
| *Coli* | Payne et al. 2019 | n.a. | - Salt (0.71 kg/m2) - Sodium bisulfate (0.71 kg/m^2^) | - *E. Coli* and Coliform were reduced in both treatments. - Note: No specifics were available. |
| *Coli* | Rathgeber et al. 2009 | - High pressure; Sodium hypochlorite 2.5%, foam; Sodium hypochlorite, 2.5%; Potassium hydroxide, 5% | - Iodine - Power Quat (quaternary ammonium compounds) | - 8.33% of Samples were positive for *E. Coli* after Iodine treatment. - The combination of any detergent with Power Quat eliminated all *E. Coli.* |
| *Coli* | Roedel et al. 2021 | (*in vitro* test) | - Formaldehyde - Chlorocresol - Quaternary ammonium compounds - Hydrogen peroxide - Peroxyacetic acid - Acetic acid | - While generally being effective, some isolates showed increased resistance against some disinfectants, including Formaldehyde and Quaternary ammonium compounds. |
| *Coli* | Sander et al. 2002 | (*in vitro* test) | - Advantage 256 1:256 (Phenol) - Poul-phene (Phenol) 1:256 - Biosentry 904 1:256 (quaternary ammonium compound) - Hydrogen peroxide 3% | - All disinfectants show the ability to eliminate the pathogen. |
| *Coli* | Shi et al. 2020 | n.a. | - Slightly acidic electrolyzed water | - An available chlorine concentration of 30mg/l or more prevents the growth of *E. Coli* isolates. - Note: If used in the barn, it showed a significant (p<0.05) reduction in total microbes, where 60mg/l, if applied via wiping, showed a 100% inactivation rate after 5 minutes in relevant disinfection areas such as floor and walls. - Note: Application by spraying is possible but less effective. |
| *Coli* | Singer et al. 2000 | - Partial litter removal; Complete litter removal | - quaternary ammonium compound, formaldehyde | - No matter the disinfection and cleaning method, a genetic fingerprint was located, indicating a repeated unsuccessful disinfection. |
| *Coli* | Soliman et al. 2009 | (*in vitro*) | - TH4 1% (Glutaraldehyde, Quaternary ammonium compounds, other) - Microzal 1% (Glutaraldehyde, Quaternary ammonium compounds) - Incospect IC 22XA 0.5% (Glutaraldehyde, Quaternary ammonium compounds, Formalin) - Povidone iodine 7.5% (other) - Formalin 2.5% | - All disinfectants eliminated *Staphylococcus* within 5 minutes in the absence of organic material. - TH4, Microzal, Formalin, and Incospect IC 22XA eliminated the pathogen in 5 minutes, even with organic matter present, while Povidone iodine took 30 minutes, but succeeded nonetheless. |
| *Coli* | Sotohy et al. 2021 | (*in vitro* test) | - Virkon S 2%, 1%, 0.5% (Potassium peroxymonosulfate) - TH5 4%, 2%, 1% (Glutaraldehyde, Quaternary ammonium compounds, other) - NP50 6%, 4%, 2% (n.a.) | - Only TH5 at the highest concentration prevents the growth of *E. Coli*. |
| *Coli* | Vasiu et al. 2014 | n.a. | n.a. | - After disinfection, some *E. Coli* isolates could be found. |
| *Salmonella* | Abdelal et al. 2016a | n.a. | - Virudox-G (n.a.); Glutarol (Glutaraldehyde); Alkadox (Sodium hypochlorite, sodium carbonate); Swift (n.a.); Iodoline (Idodine, n.a.); Phenodex (Phenol, n.a.). All et 0.5%, 1%. | - At 0.5% Virudox-G was the only disinfectant to achieve a complete disinfection, while Phenodex didn´t even achieve the 99% mark. Virudox-G, Glutarol, and Alkadox achieved full disinfection at a concentration of 1%; the rest remained at 99.9% or 99.8%. |
| *Salmonella* | Abdelal et al. 2016b | - Farm B: Biosafe 3% | - Farm A: phenol 5%, spayed - Farm B: Virocide 0.5% (Glutaraldehyde and Quaternary ammonium), sprayed | - 8.33% of relevant samples from farm A were positive for *Salmonella*, while 0.083% of relevant samples were positive across four serotypes of *Salmonella* in farm B. |
| *Salmonella* | Abd-Elall et al. 2023 | (*in vitro* test) | - Hydrogen peroxide - Sodium hypochlorite - Virkon S (Potassium peroxymonosulfate); Glutaraldehyde - Copper sulphate - All at 1%, 2%,5%. | - Except for copper sulphate, all disinfectants eliminated the biofilm at a concentration of 5%. - Except for Vikron S, a concentration of 2% is also enough. |
| *Salmonella* | Aksoy et al. 2020 | (*in vitro* test) | - Quaternary ammonium compounds - Iodine - Hydrogen peroxide - Glutaraldehyde - All at 1%, 0.5% and 0.00025%. | - Quaternary ammonium compounds at a concentration of 1% and 0.5% were 100% effective, as well as 0.0025% at a temperature of 20 degrees Celsius. - Iodine was also at a concentration of 1% and 0.5% 100% effective, but at 0.0025% only against some serotypes. - Glutaraldehyde and Hydrogen peroxide are effective even at 0.00025% and at 4 degrees Celsius. |
| *Salmonella* | Badr and Yoseif 2014 | - B: Degaclean 51 5-10ml/l applied by foam | - A: Formalin 3.7% - B: Peraclean 15 (Other) 3ml/l | - The pathogen can be found in samples after disinfection from A, but not B. |
| *Salmonella* | Bassani et al. 2021 | (*in vitro* test) | - Sodium hypochlorite 0.5%, 1.0%, Quaternary ammonium compounds 100ppm, 200ppm | - While the results from the 1.0% concentration test are unknown, only 10% of *Salmonella* strains survived the 0.5% sodium hypochlorite treatment. - Quaternary ammonium compounds with 100ppm were ineffective against up to 40% of Strains, depending on temperature, and still against up to 10% with 200 ppm. |
| *Salmonella* | Bezek et al. 2023 | (*in vitro* test) | - Ecocid® S (other); Ethanol; Hydrogen peroxide | - All disinfectants are effective. |
| *Salmonella* | Cardinale et al. 2004 | n.a. | n.a. | - The lack of detergent is a risk factor (OR: 6.16) |
| *Salmonella* | Castañeda-Gulla et al. 2020 | - Blown, high-pressure, Biostrip 3000 | - Glutasan QCT (Glutaraldehyde and Quaternary ammonium compounds), high-pressure | - Depending on the cycle and location, between 0 and 30% were positive after disinfection. |
| *Salmonella* | Course et al. 2021 | n.a. | n.a. | - Less contamination on dry-cleaned concrete floors than on wet-cleaned wooden floors. - Lower counts were observed several days after disinfection. |
| *Salmonella* | Drauch et al. 2020 | (*in vitro* test) | - ALDEKOL DES® 04 2.0% (Aldehyde and quarternary ammonium compounds) - Calgonit DS 680 2.0% (Aldehyde and quarternary ammonium compounds) - Calgonit Sterizid P12 DES 2.0% (Aldehyde and quarternary ammonium compounds) - DESINTEC® FL-des GA forte 2.5% (Aldehyde and quarternary ammonium compounds) - ROTIE-CID F1 1.0% (Aldehyde and quarternary ammonium compounds) - DESINTEC® Peroxx Liquid 0.50% (Peroxygen compounds) - ROTIE-PER Spezial 1.0% (Peroxygen compounds) - Virkon™ S 1.0% (Peroxygen compounds) - Calgonit Sterizid ECOKOK 0.5% (Cresol) - Profex 99 1.5% (Alkylamines) | - Calgonit sterizid P12 DES, DESINTEC® FL-des GA forte, and Calgonit sterizid ECOKOK were able to achieve a 100% bacteriostatic effect at the recommended concentration. Profex 99 didn´t succeed even at 4 times the recommended concentration. - Note: Only 7 of 10 disinfectants were presented. - Aldehydes and quaternary ammonium compounds had the best bactericidal efficacy with 84.29%. Cresol and alkylamines exhibit a relatively small effect, with 58.93% and 50.0%, respectively. |
| *Salmonella* | Moustafa Gehan et al. 2009 | (*in vitro* test) | - Perasan® 1% (Peracetic acid, H2O2, other) - H2O2® 3% (H2O2, other); Aldekol® 1% (Glutaraldehyde, Quaternary ammonium compounds, Formalin) - Quatovet® 1% (Quaternary ammonium compounds); - Virkon S® 1% (Potassium peroxymonosulfate) | - All disinfectants, except Virkon-S, were effective against the pathogen after 30 minutes. |
| *Salmonella* | Gradel and Rattenborg 2003 | n.a. | n.a. | - Surface-and-pulse fog disinfection is more effective in preventing infection with multiple *Salmonella* types than those alone. |
| *Salmonella* | Gradel et al. 2005 | (*in vitro* test) | - Formaldehyde - Bio Komplet Plus (Glutaraldehyde, Quaternary ammonium compounds) - Virkon S (Potassium-peroxymonosulfate) - Farm Fluid S (phenol) - FAM 30 (Iodophor) | - Farm Fluid S is the only disinfectant that doesn’t significantly impact *Salmonella* (p>0.05). - There is a high difference between the *Salmonella* strains and their sensitivity to the disinfectants. |
| *Salmonella* | Higgins et al. 1982 | n.a. | n.a. | - 6 out of 9 Farms remained positive after cleaning and disinfection. |
| *Salmonella* | Kaoud and Yosseif 2013 | (*in vitro* test) | - White wash 20% (Other) - Formalin 4% (Formaldehyde) - Iodophor 1% - Phenuique 3% (Other) - Envirolyte-Anolyte 1\500 (sodium hypochlorite, other). - Additional: silver nanoparticles. | - While all disinfectants significantly reduced the amount of pathogen (p<0.05), Envirolyte-Anolyte (1\500) was the only disinfectant to effectively eliminate all *Salmonella*. - With the addition of silver nanoparticles, the disinfection effect was significantly improved (p<0,05), and Formalin 4% as well as Phenuique 3% completely removed the pathogen. |
| *Salmonella* | Kloska et al. 2017a | - routine cleaning | - routine disinfection | - 46.67% of the samples were positive after the usual cleaning and disinfection. |
| *Salmonella* | Kloska et al. 2017b | - Dry cleaned + multiple times with 60-degree water + commercial foam | - A: peracetic acid - B: Formalin, fogging. Additionally, sodium hypochlorite. | - Both showed 100% effectiveness after sodium hypochlorite usage. |
| *Salmonella* | Kranjc et al. 2024 | - (*in vitro* test) | - Calgonit Sterizid P12 DES (glutaraldehyde, QACs) - DioksiLEK® (chlorine dioxide solution); Interkokask® (chlorocresol) - Electrolyzed water; Virocid® (quaternary ammonium, glutaraldehyde, isopropanol) | - All disinfectants are effective. - DioksiLEK® needed to far exceed the recommended concentration of 0.2 – 1% to 3.5% to reach the minimal inhibitory concentrations. - Interkokask® significantly reduces *Salmonella* with Biofilm, in the presence of Polystyrene, and if improperly stored. |
| *Salmonella* | Lahellec et al. 1986 | - Washed + sweeping | - Phenol, spray - Formaldehyde, fumigation | - Only 2 out of 10 farms didn’t have *Salmonella*-positive samples. |
| *Salmonella* | Le Bouquin et al. 2010 | n.a. | n.a. | - Removal of equipment before disinfection reduces *Salmonella*-positive Flocks (p<0.05). - Neither a second disinfectant nor the application method of said second disinfectant is statistically significant (p>0.05). |
| *Salmonella* | Luyckx et al 2015b | - high pressure, soap - KenoTM San 1% (Sodium hydroxide) - Intra Power Foam 3% (Sodium hydroxide) - Sodium Hydroxide 50% 1% (Sodium hydroxide) - Ino Net 3% (Sodium hydroxide + potassium hydroxide) - Protocol 1: Overnight soaking and cleaning with warm water - Protocol 2: Overnight soaking and cleaning with cold water - Protocol 3: Cleaning with warm water - Protocol 4: Cleaning with cold water | - Cid 20 2% (Glutaraldehyde, Quaternary ammonium compounds, alcohols), fogging - Desbest 700 1% (Glutaraldehyde, Quaternary ammonium compounds, alcohols), spraying - ViroCid 1.8% (Glutaraldehyde, Quaternary ammonium compounds, alcohols), spraying - Cid 20 2% (Glutaraldehyde, Quaternary ammonium compounds, alcohols), spraying - Hyprelva SL 2% (Glutaraldehyde, Quaternary ammonium compounds, alcohols), fogging | - Only on one of the four farms*, Salmonella* could be found, with slightly below 10% samples being positive, and reduced to only two positive samples following disinfection, resulting in a reduction of 71.82%. |
| *Salmonella* | Marin et al. 2009 | - n.a. | - Glutaraldehyde (50% vol/vol) - Formaldehyde (37% vol/vol) - Hydroxide peroxide (35% vol/vol) - All at 1%. | - Glutaraldehyde eliminated 30% of strains, Formaldehyde and hydrogen peroxide less than 7% |
| *Salmonella* | Marin et al. 2011 | - pressure washer | - Glutaraldehyde Formaldehyde–Glutaraldehyde compound | - 10.8% of broiler houses had positive samples on surfaces after cleaning and disinfection |
| *Salmonella* | Newton et al. 2020 | - cleaned, power-washed | - Glutaraldehyde, Formaldehyde, Formalin, high pressure | - The glutaraldehyde-formaldehyde-formalin combination prevented carryover. |
| *Salmonella* | Newton et al. 2021 | n.a. | n.a. | - Only 0.01% of samples were positive after cleaning and disinfection. |
| *Salmonella* | Payne et al. 2005 | n.a. | - Phenol - Potassium peroxymonosulfate - Nascent oxygen - Quaternary ammonia - Same concentration different application amount: (≈0.108l /m^2^; ≈1.345l/m^2^) | - The low and high application amounts were unsuccessful in eliminating all the *Salmonella*. |
| *Salmonella* | Rose et al. 2000 | n.a. | n.a. | - Disinfection by Farm personnel instead of contractors significantly increases the risk of *Salmonella* infection (OR 3.9, p<0.05). - Using only one or no disinfectant in comparison to two increases the risk significantly (OR 13.3/31, p<0.05) |
| *Salmonella* | Rose et al. 2003 | n.a. | n.a. | - Waiting for 5 days or more after cleaning increases the risk of *Salmonella* infection in the following flock (OR: 4.7) |
| *Salmonella* | Sander et al. 2002 | n.a. | - Advantage 256 1:256 (Phenol) - Poul-phene (Phenol) 1:256 - Biosentry 904 1:256 (quaternary ammonium compound) - Hydrogen peroxide 3% | - All disinfections show the ability to eliminate the pathogen. |
| *Salmonella* | Sevilla-Navarro et al. 2024 | - Cleaning with detergent | - Glutaraldehyde - Quaternary ammonium compounds - Peroxides - Additionally bacteriophages | - Phage treatment resulted in a reduction from 100% to 36% positive *Salmonella* samples in the barn after two applications. - All Pathogens were eliminated after the disinfection. |
| *Salmonella* | Shi et al. 2020 | - n.a. | - Slightly acidic electrolyzed water | - Available chlorine concentration of 20mg/l or more prevents the growth of *Salmonella* isolates. - Note: If used in the barn, it showed a significant (p<0.05) reduction in total microbes, where 60mg/l, if applied via wiping, showed a 100% inactivation rate after 5 minutes in relevant disinfection areas such as floor and walls. - Note: Application by spraying is possible but less effective. |
| *Salmonella* | Sotohy et al. 2021 | - (*in vitro* test) | - Virkon S 2%, 1%, 0.50% (Potassium peroxymonosulfate) - TH5 4%, 2%, 1% (Glutaraldehyde, Quaternary ammonium compounds, other) - NP50 6%, 4%, 2% (n.a.) | - While all disinfectants showed a reduction, only TH5 at the highest concentration was able to prevent growth. |
| *Salmonella* | Sperandio et al. 2023 | - (*in vitro* test) | - Thymus vulgaris essential oil - Monoterpene thymol | - Thymus vulgaris essential oil showed a significant (p<0.05) effect in preventing growth at a concentration of 0.1% or 0.2% depending on the Isolate. - Monoterpene thymol proved ineffective against one isolate under the tested concentrations, while the others were at 0.094% or less. |
| *Salmonella* | Stringfellow et al. 2009 | - (*in vitro* test) | - Quaternary ammonium compounds - Phenol - Chlorhexidine - Binary compound (Quaternary ammonium compounds) | - All significantly reduced the pathogens (p<0.05). No disinfectant was able to successfully eliminate the pathogen when organic matter was introduced. |
| *Staphylococcus* | Abdelal et al. 2016a | n.a. | - Virudox-G (Glutaraldehyde, QAC) - Glutarol (Glutaraldehyde) - Alkadox (Sodium hypochlorite, sodium carbonate) - Swift (n.a.) - Iodoline (Iodine) - Phenodex (Phenol) - All et 0.50%, 1%. | - At 0.50% Virudox-G and Glutarol resulted in a complete reduction, while the rest achieved an effectiveness of 99.7% to 99.9%. - At 1% Iodoline and Phenodex only achieved an effectiveness of 99.9%, while the rest eliminated the pathogen |
| *Staphylococcus* | Abdelal et al. 2016b | - Farm B: Biosafe 3% | - Farm A: phenol 5%, spayed - Farm B: Virocide 0.50% (Glutaraldehyde and Quaternary ammonium), sprayed | - 20% of relevant samples from Farm A were positive for Staphylococcus, while 0.33 % from Farm B were. |
| *Staphylococcus* | Abd-Elall et al. 2023 | (*in vitro* test) | - Hydrogen peroxide; Sodium hypochlorite - Virkon S (Potassium peroxymonosulfate) - Glutaraldehyde - Copper sulphate - All at 1%, 2%, 5%. | - Hydrogen peroxide and sodium hypochlorite are most effective in eliminating *Staphylococcus* at a concentration of 5%. - Glutaraldehyde and especially copper sulphate, while reducing the amount somewhat, are less effective |
| *Staphylococcus* | Abou-Khadra et al. 2024 | (*in vitro* test) | - TH4 ® 2%, 1%, 0.50%, 0.25% (Glutaraldehyde, Quaternary ammonium compounds, other) | - *Staphylococcus* is susceptible to TH4 ®. |
| *Staphylococcus* | Aidaros et al. 2022 | (*in vitro* test) | - Prophyl 2000® 0.40%, 2%, 4% (Glutaraldehyde, Quaternary ammonium compounds, Phenol) - G7® 0.25% 0.50%,1% (Glutaraldehyde, Quaternary ammonium compounds) - Pron-Tech® 0.10%, 0.20%, 0.50% (Quaternary ammonium compounds, urea); Alkadox® 0.50%, 1%, 1.5% (Sodium hypochlorite, sodium carbonate) - Biodine® 0.50%, 1%, 1.5% (Iodine, other) | - Prophyl 2000® can eliminate all *Staphylococcus* within 90 minutes or less, even in the presence of organic material within their recommended concentration. - G7®, Pron-Tech®, and Alkadox® needed to surpass their recommended concentration to achieve total disinfection in the presence of organic material. - Biodine® failed in achieving successful disinfection even in higher concentrations |
| *Staphylococcus* | Badr and Yoseif 2014 | - B: Degaclean 51 5-10ml/l applied by foam | - A: Formalin 3.7% - B: Peraclean 15 (H2O2, Peratic acid) 3ml/l | - *Staphylococcus* can be found in samples after disinfection from A, but not B |
| *Staphylococcus* | Castañeda-Gulla et al. 2020 | - Blown, high-pressure, Biostrip 3000 | - Glutasan QCT (Glutaraldehyde and Quaternary ammonium compounds), high-pressure | - The pathogen was not significantly reduced. (p > 0.05) |
| *Staphylococcus* | Elsayed et al. 2020 | (*in vitro* test) | - DC&R® 4%, 8%, 16% (Formaldehyde, Quaternary ammonium compounds, Other) - Virkon S® 0.50%, 1%, 2% (Potassium peroxymonosulfate) - TH4++ 0.25%, 0.50%, 1% (Glutaraldehyde, Quaternary ammonium compounds, other) - Tek-Trol 0.25%, 0.50%, 1% (Phenol) - Peracetic acid 0.25%, 0.50%, 1%. - Additionally, silver- as well as copper nanocomposites | - DC&R®, Teck-Trol, and peracetic acid achieved a 99.99% effectiveness in high concentration (16%, 1%, 1%). - The nanocomposites help to eliminate all *Staphylococcus* with medium or high concentration, except the combination of silver nanocomposites with Tek-Trol. |
| *Staphylococcus* | Moustafa Gehan et al. 2009 | (*in vitro* test) | - Perasan® 1% (Peracetic acid, H2O2, other) - H2O2® 3% (H2O2, other) - Aldekol® 1% (Glutaraldehyde, Quaternary ammonium compounds, Formalin) - Quatovet® 1% (Quaternary ammonium compounds) - Virkon S® 1% (Potassium peroxymonosulfate) | - All disinfectants were effective against the pathogen after 30 minutes. |
| *Staphylococcus* | Li et al. 2019 | n.a. | - Aldehydes - Alcohol - Quaternary ammonium salt | - *Staphylococcus* can survive disinfection in Particulate matter less than 2.5 μm. |
| *Staphylococcus* | Payne et al. 2019 | n.a. | - Salt (0.71 kg/m^2^) - Sodium bisulfate (0.71 kg/m^2^) | - With the use of sodium bisulfate, no sample after 72h was positive. - Salt is ineffective. |
| *Staphylococcus* | Sander et al. 2002 | (*in vitro* test) | - Advantage 256 1:256 (Phenol) - Poul-phene (Phenol) 1:256 - Biosentry 904 1:256 (Quaternary ammonium compound) - Hydrogen peroxide 3% | - All disinfectants show the ability to eliminate the pathogen. |
| *Staphylococcus* | Shi et al. 2020 | n.a. | - Slightly acidic electrolyzed water | - An available chlorine concentration of 20mg/l or more prevents the growth of *Staphylococcus* isolates. - Note: If used in the barn, it showed a significant (p<0.05) reduction in total microbes, where 60mg/l, if applied via wiping, showed a 100% inactivation rate after 5 minutes in relevant disinfection areas such as floor and walls. - Note: Application by spraying is possible but less effective. |
| *Staphylococcus* | Soliman et al. 2009 | (*in vitro*) | - TH4 1% (Glutaraldehyde, Quaternary ammonium compounds, other) - Microzal 1% (Glutaraldehyde, Quaternary ammonium compounds) - Incospect IC 22XA 0.50% (Glutaraldehyde, Quaternary ammonium compounds, Formalin) - Povidone idodine 7.5% (Iodine) - Formalin 2.5% | - All disinfectants eliminated *Staphylococcus* within 5 minutes in the absence of organic material. - Microzal and Incospect IC 22XA eliminated the pathogen in 5 minutes, even with organic matter present, while the rest took up to 30 minutes, but all succeeded nonetheless. |
| *Staphylococcus* | Sotohy et al. 2021 | (*in vitro* test) | - Virkon S 2%, 1%, 0.5% (Potassium peroxymonosulfate) - TH5 4%, 2%, 1% (Glutaraldehyde, Quaternary ammonium compounds, other) - NP50 6%, 4%, 2% (n.a.) | - Virkon S as well as TH5 prevented the growth of the pathogen with the recommended concentration of 1% and 2% respectively. - NP50 was not successful in preventing the growth at a concentration of 4%, but if it´s increased to 6%. |
| *Staphylococcus* | Stringfellow et al. 2009 | (*in vitro* test) | - QAC (Quaternary ammonium compounds) - Phenol - Chlorhexidine - Binary compound (Quaternary ammonium compounds) | - All significantly reduced *Staphylococcus* (p<0.05). Chlorhexidine has little to no effect when introduced to pathogens in the presence of organic matter, while the binary, as well as the QAC, loses effectiveness. - Phenol showed no reduced effectiveness in the presence of organic matter. - Besides phenol, which showed no sign of decreased effectiveness, and chlorhexadine, which remained ineffective, the other two disinfectants showed reduced effectiveness when stored for 30 weeks in comparison to being freshly made. |
| Other (Total) | Fate et al. 1985 | n.a. | - Aldacide (Formaldehyde) - DC&R (quaternary ammonium compound, Formaldehyde, other) - Iofec-20 (Iodophor) - Pantek II (phenols, cresols, other). | - Bacteria can be found no matter the disinfectant. |
| Other (Pasteurella multocida) | Sander et al. 2002 | (*in vitro* test) | - Advantage 256 1:256 (Phenol) - Poul-phene (Phenol) 1:256 - Biosentry 904 1:256 (quaternary ammonium compound) - Hydrogen peroxide 3% | - All disinfectants show the ability to eliminate the pathogen. |
| Other (*Proteus mirabilis*) | Sander et al. 2002 | n.a. | - Advantage 256 1:256 (Phenol) - Poul-phene 1:256 (Phenol) - Biosentry 904 1:256 (quaternary ammonium compound) - Hydrogen peroxide 3% | - All disinfections show the ability to eliminate the pathogen. |
| Other (*Pseudomonas diminuta*) | Sander et al. 2002 | n.a. | - Advantage 256 1:256 (Phenol) - Poul-phene 1:256 (Phenol) - Biosentry 904 1:256 (quaternary ammonium compound) - Hydrogen peroxide 3% | - All disinfections show the ability to eliminate the pathogen. |
| Other (*Pseudomonas aeruginosa*) | Sander et al. 2002 | n.a. | - Advantage 256 1:256 (Phenol) - Poul-phene 1:256 (Phenol) - Biosentry 904 1:256 (quaternary ammonium compound) - Hydrogen peroxide 3% | - All disinfections show the ability to eliminate the pathogen. |
| Other (*Enterococcus faecalis*) | Sander et al. 2002 | n.a. | - Advantage 256 1:256 (Phenol) - Poul-phene 1:256 (Phenol) - Biosentry 904 1:256 (quaternary ammonium compound) - Hydrogen peroxide 3% | - All disinfections show the ability to eliminate the pathogen. |
| Other (Aerobic Bacteria) | Payne et al. 2005 | n.a. | - Phenol - Potassium peroxymonosulfate - Nascent oxygen - Quaternary ammonia - Same concentration different application amount: (≈0.108l /m^2^; ≈1.345l/m^2^) | - While dependent on the sample time, the disinfectants can significantly (p<0.05) reduce the number of aerobic bacteria. |
| Other (Aerobic Bacteria) | Ward et al. 2006 | - A: Hot water, B: Blown | - Virkon (Potassium peroxymonosulfate) 1% | - Aerobic Bacteria count was not significantly affected by the cleaning. (p>0.05) |
| Other (*Enterobacteriaceae*) | Ward et al. 2006 | - A: Hot water, B: Blown | - Virkon (Potassium peroxymonosulfate) 1% | - The amount of *Enterobacteriaceae* was significantly reduced by the cleaning (p<0.05) |
| Other (*Enterococcus faecium*) | Garcia-Migura et al. 2007 | - High pressure | - Quaternary ammonium compounds, peracetic acid, and hydrogen peroxide | - All selected places had positive samples, depending on location, up to 50% of the samples were positive. |
| Other (Aerobic Bacteria) | Rathgeber et al. 2009 | - High pressure - Sodium hypochlorite 2.5%, foam - Sodium hypochlorite, 2.5%, potassium hydroxide, 5% | - Iodine | - Aerobic Bacteria count could be reduced but not eliminated. |
| Other (*Pseudomonas aeruginosa*) | Soliman et al. 2009 | (*in vitro*) | - TH4 1% (Glutaraldehyde, Quaternary ammonium compounds, other) - Microzal 1% (Glutaraldehyde, Quaternary ammonium compounds) - Incospect IC 22XA 0.5% (Glutaraldehyde, Quaternary ammonium compounds, Formalin) - Povidone idodine 7.5% (Iodine, other) - Formalin 2.5% | - All disinfectants eliminated the pathogen in the presence of organic material in 30 minutes or less. |
| Other (*Klebsiella pneumoniae*) | Soliman et al. 2009 | (*in vitro*) | - TH4 1% (Glutaraldehyde, Quaternary ammonium compounds, other) - Microzal 1% (Glutaraldehyde, Quaternary ammonium compounds) - Incospect IC 22XA 0.5% (Glutaraldehyde, Quaternary ammonium compounds, Formalin) - Povidone idodine 7.5% (Iodine, other) - Formalin 2.5% | - All disinfectants eliminated the pathogen in the presence of organic material in 30 minutes or less. |
| Other (*Pseudomonas aeruginosa*) | Moustafa Gehan et al. 2009 | (*in vitro* test) | - Perasan® 1% (Peracetic acid, H2O2, other) - H2O2® 3% (H2O2, other); Aldekol® 1% (Glutaraldehyde, Quaternary ammonium compounds, Formalin) - Quatovet® 1% (Quaternary ammonium compounds) - Virkon S® 1% (Potassium peroxymonosulfate). | - Virkon S® was the only disinfectant to generally not prevent the growth of *Pseudomonas aeruginosa*, while Quatovet® also failed in the presence of organic material. |
| Other (*Listeria monocytogenes*) | Aury et al. 2011 | n.a. | n.a. | - Thermal fogging in comparison to spraying increases the risk of *Listeria monocytogenes* flocks significantly (p< 0.05, OR 3.76) |
| Other (*Clostridium perfringens*) | Engström et al 2012. | - High pressure, Detergent | - Neopredisan® (cresol) - Virocid® (Quaternary ammonium compounds, Glutaraldehyde, other) | - 36.46% of samples were positive before the placement of the chicks. |
| Other (*Enterococcus faecium*) | Nilsson et al. 2013 | n.a. | - A: Glutaraldehyde - B: Chloride - C: Formalin | - Formalin can eliminate *Enterococcus faecium*, while Glutaraldehyde and Chlorine fail, with 20% and 50% of samples being positive, respectively. |
| Other (*Proteus mirabili*) | Vasiu et al. 2014 | n.a. | n.a. | - *Proteus mirabilis* was present after disinfection. |
| Other (*Pseudomonas aeruginosa*) | Badr and Yoseif 2014 | - B: Degaclean 51 5-10ml/l applied by foam | - A: Formalin 3.7% - B: Peraclean 15 (Other). 3ml/l | - Formalin didn´t fully eliminate a lot of pathogens, including *Pseudomonas aeruginosa, while after B,* only *Bacillus* *spp.* could be found. |
| Other (Total colony count) | Badr and Yoseif 2014 | - B: Degaclean 51 5-10ml/l applied by foam | - A: Formalin 3.7% - B: Peraclean 15 (Other). 3ml/l | - The total colony court was reduced by over 99.99%, but never fully eliminated. |
| Other (*Clostridium perfringens*) | Nasr et al. 2014 | - Fairy 1% (Foaming agent + surfactant); Urea 1% | - Calcium hypochlorite 5%; Glutaraldehyde 1% | - Despite Calcium hypochlorite performing better with both cleaning agents together, neither combination was able to eliminate all *Clostridium perfringens*. |
| Other (Total microbial counts) | Burbarelli et al. 2015 | - A: low pressure, detergent 4%. - B: high-pressure, alkaline detergent 4% | - B: glutaraldehyde, formaldehyde 0.4%, para-chlor-meta-cresol 4% | - Total microbial counts are reduced but not eliminated. While B showed a higher reduction in total microbial count compared to A in the case of reused litter, the data with new litter is inconclusive due to vastly different colony counts pre-disinfection. |
| Other (Total aerobic flora) | Luyckx et al. 2015a | - Varying see Luyckx et al 2015b | - Cid 20 2% (Glutaraldehyde, Quaternary ammonium compounds, alcohols), fogging - Desbest 700 1% (Glutaraldehyde, Quaternary ammonium compounds, alcohols), spraying | - The total aerobic flora, while being reduced, couldn´t be fully eradicated by either protocol. |
| Other (*Enterococcus spp.)* | Luyckx et al. 2015a | - Varying see Luyckx et al 2015b | - Cid 20 2% (Glutaraldehyde, Quaternary ammonium compounds, alcohols), fogging - Desbest 700 1% (Glutaraldehyde, Quaternary ammonium compounds, alcohols), spraying | - *Enterococcus spp.*, while being reduced, couldn´t be fully eradicated by either protocol. |
| Other (Total aerobic flora) | Luyckx et al 2015b | - high pressure, soap KenoTM San 1% (Sodium hydroxide) - Intra Power Foam 3% (Sodium hydroxide) - Sodium Hydroxide 50% 1% (Sodium hydroxide) - Ino Net 3% (Sodium hydroxide + potassium hydroxide) - Protocol 1: Overnight soaking and cleaning with warm water - Protocol 2: Overnight soaking and cleaning with cold water - Protocol 3: Cleaning with warm water - Protocol 4: Cleaning with cold water | - Cid 20 2% (Glutaraldehyde, Quaternary ammonium compounds, alcohols), fogging - Desbest 700 1% (Glutaraldehyde, Quaternary ammonium compounds, alcohols), spraying - ViroCid 1.8% (Glutaraldehyde, Quaternary ammonium compounds, alcohols), spraying - Cid 20 2% (Glutaraldehyde, Quaternary ammonium compounds, alcohols), spraying - Hyprelva SL 2% (Glutaraldehyde, Quaternary ammonium compounds, alcohols), fogging | - The total aerobic flora, while being reduced, couldn´t be fully eradicated by either protocol. |
| Other (*Enterococcus* *spp.*) | Luyckx et al 2015b | - high pressure, soap KenoTM San 1% (Sodium hydroxide) - Intra Power Foam 3% (Sodium hydroxide) - Sodium Hydroxide 50% 1% (Sodium hydroxide) - Ino Net 3% (Sodium hydroxide + potassium hydroxide) - Protocol 1: Overnight soaking and cleaning with warm water - Protocol 2: Overnight soaking and cleaning with cold water - Protocol 3: Cleaning with warm water - Protocol 4: Cleaning with cold water | - Cid 20 2% (Glutaraldehyde, Quaternary ammonium compounds, alcohols), fogging - Desbest 700 1% (Glutaraldehyde, Quaternary ammonium compounds, alcohols), spraying - ViroCid 1.8% (Glutaraldehyde, Quaternary ammonium compounds, alcohols), spraying - Cid 20 2% (Glutaraldehyde, Quaternary ammonium compounds, alcohols), spraying - Hyprelva SL 2% (Glutaraldehyde, Quaternary ammonium compounds, alcohols), fogging | - *Enterococcus* *spp.*, while being reduced, couldn´t be fully eradicated by either protocol. |
| Other (Total bacteria) | Bashandy et al. 2016 | - A: Surfactant, Detergent - B: Surfactant, Detergent, Foam. - C: Surfactant, Detergent, Bleaching powder (Calcium hypochlorite) | - A: Quicklime (Other), Cresolic acid. - B: Quicklime (Other), Bleaching powder (Calcium hypochlorite). - C: Quicklime (Other), Cresolic acid, Bleaching powder (Calcium hypochlorite). | - No bacteria could be found on the surfaces 24 hours after any program. |
| Other (*Enterobacteriaceae*) | Luyckx et al. 2017 | - foaming cleaning, Keno™san 1% (Sodium hydroxide) | - D50 (Hydrogen peroxide and Peroxyacetic acid) | - All *Enterobacteriaceae* died at a concentration of 1% D50 or less, depending on the specific kind. |
| Other (*Enterococcus spp.*) | Luyckx et al. 2017 | - foaming cleaning, Keno™san 1% (Sodium hydroxide) | - D50 (Hydrogen peroxide and Peroxyacetic acid) | - For *Enterococcus*, a concentration of 0.0625% or less, depending on the strain, D50 is enough to eliminate it. |
| Other (varying) | Jiang et al. 2018 | n.a | - Ozone 100 g/h, 1h; Available chlorine 1:1500, spray; Quaternary ammonium salt 1:2000, spray; Glutaraldehyde 1:1000, spray; Aldehydes, quaternary ammonium salt, alcohol, 1:1500, spray | - Chlorine has the least impact. No disinfectant was able to eliminate all bacteria completely. |
| Other (Aerobic bacteria) | Payne et al. 2019 | n.a. | - Salt (0.71 kg/m^2^) - Sodium bisulfate (0.71 kg/m^2^) | - With the use of sodium bisulfate, the total amount of aerobic bacteria was reduced, but not wiped out. |
| Other (Varying) | Li et al. 2019 | n.a. | - Aldehydes - Alcohol - Quaternary ammonium salt | - A variety of pathogens, including Pseudomonas, can survive disinfection in particles. |
| Other (Total aerobic counts) | Mateus-Vargas et al. 2022 | - Hot water, high pressure | - Aldekol Des® 03 3%-5%, sprayed, 5%-8%, fogged (Glutaraldehyde, Formaldehyde, QAC) | - While disinfection significantly (p<0.05) reduced the total aerobic bacteria, it couldn´t eliminate them. |
| Other (*Enterococcus spp.*) | Mateus-Vargas et al. 2022 | - Hot water, high pressure | - • Aldekol Des® 03 3%-5%, sprayed, 5%-8%, fogged (Glutaraldehyde, Formaldehyde, QAC) | - While disinfection significantly (p<0.05) reduced *Enterococcus* *spp.*, it couldn´t eliminate them. |
| Other (*Enterobacteriaceae spp.)* | Mateus-Vargas et al. 2022 | - Hot water, high pressure | - Aldekol Des® 03 3%-5%, sprayed, 5%-8%, fogged (Glutaraldehyde, Formaldehyde, QAC) | - While disinfection significantly (p<0.05) reduced *Enterobacteriaceae* *spp.*, it couldn´t eliminate them. |
| Other (*Pasteurella multocida*) | Aidaros et al. 2022 | (*in vitro* test) | - Prophyl 2000® 0.40%, 2%, 4% (Glutaraldehyde, Quaternary ammonium compounds, Phenol) - G7® 0.25% 0.50%,1% (Glutaraldehyde, Quaternary ammonium compounds) - Pron-Tech® 0.10%, 0,2%, 0.50% (Quaternary ammonium compounds, urea) - Alkadox® 0.50%, 1%, 1.5% (Sodium hypochlorite, sodium carbonate) - Biodine® 0.50%, i0.50%, 1%, 1.5%, 1%, 1.5% (Iodine, other) | - Only Prophyl 2000® and G7® were able to eliminate *Pasteurella multocida* in the absence as well as the presence of organic matter within the recommended doses. - Only Biodine® fails to eliminate the pathogen in the presence of organic matter at the highest concentration. |
| Other (*Pseudomonas aeruginosa*) | Ibrahim et al. 2023 | - high pressure, Bioshield 3000 | - Bioshield 1% (Glutaraldehyde, quaternary ammonium compound) - Bioshield P 1% (Glutaraldehyde, quaternary ammonium, compound) - Bi-OO-Cyst 0.625% (other) | - Despite having the same procedure, one house was completely free of *Pseudomonas*, while the others have still some remaining. |
| Other (*Proteus mirabili*) | Abou-Khadra et al. 2024 | (*in vitro* test) | - TH4 ® 2%, 1%, 0.50%, 0.25% (Glutaraldehyde, Quaternary ammonium compounds, other) | - *Proteus mirabili* is susceptible to TH4®. |
| Other (*Pseudomonas aeruginosa*) | Abou-Khadra et al. 2024 | (*in vitro* test) | - TH4 ® 2%, 1%, 0.5%, 0.25% (Glutaraldehyde, Quaternary ammonium compounds, other) | - *Pseudomonas aeruginosa* is susceptible to TH4®. |
| Other (*Enterococcus cecorum*) | Tessin et al. 2024 | n.a. | - A: Peracetic acids B: Aldehydes | - In Farm A 10% of samples were positive for *Enterococcus cecorum*, while only 3.33% in Farm B. |

# References Cited Only in Supplementary Material

Aury, K., S. Le Bouquin, M.-T. Toquin, A. Huneau-Salaün, Y. Le Nôtre, V. Allain, I. Petetin, P. Fravalo, and M. Chemaly. 2011. Risk factors for *Listeria monocytogenes* contamination in French laying hens and broiler flocks. Prev. Vet. Med. 98:271-278. <https://doi.org/10.1016/j.prevetmed.2010.11.017>

Bashandy, E., S. Wanis, S. Nasr, M. Abdelaty, and O. Zahran. 2016. Assessment of disinfectant performance procedures applied in small sector of Egyptian poultry farms. Vet. Med. J. (Giza) 62:11-19. <https://vmjg.journals.ekb.eg/article_363153.html>

Berndtson, E., U. Emanuelson, A. Engvall, and M.-L. Danielsson-Tham. 1996. A 1-year epidemiological study of campylobacters in 18 Swedish chicken farms. Prev. Vet. Med. 26:167-185. <https://doi.org/10.1016/0167-5877(95)01008-4>

Bezek, K., J. Avberšek, O. Zorman Rojs, and D. Barlič-Maganja. 2023. Antimicrobial and antibiofilm effect of commonly used disinfectants on *Salmonella* Infantis isolates. Microorganisms 11:301. <https://doi.org/10.3390/microorganisms11020301>

Burbarelli, M. F. C., C. E. B. Merseguel, P. A. P. Ribeiro, K. D. Lelis, G. V. Polycarpo, A. C. P. Carão, R. A. Bordin, A. M. Fernandes, R. L. M. Souza, M. E. G. Moro, and R. Albuquerque. 2015. The effects of two different cleaning and disinfection programs on broiler performance and microbiological status of broiler houses. Braz. J. Poult. Sci. 17:575-580. <https://doi.org/10.1590/1516-635X1704575-580>

Course, C. E., P. Boerlin, D. Slavic, J.-P. Vaillancourt, and M. T. Guerin. 2021. Factors associated with *Salmonella enterica* and *Escherichia coli* during downtime in commercial broiler chicken barns in Ontario. Poult. Sci. 100:101065. <https://doi.org/10.1016/j.psj.2021.101065>

Engström, B. E., A. Johansson, A. Aspan, and M. Kaldhusdal. 2012. Genetic relatedness and *netB* prevalence among environmental *Clostridium perfringens* strains associated with a broiler flock affected by mild necrotic enteritis. Vet. Microbiol. 159:260-264. <https://doi.org/10.1016/j.vetmic.2012.03.024>

Evans, S. J., and A. R. Sayers. 2000. A longitudinal study of *Campylobacter* infection of broiler flocks in Great Britain. Prev. Vet. Med. 46:209-223. <https://doi.org/10.1016/s0167-5877(00)00143-4>

Fate, M. A., J. K. Skeeles, C. E. Whitfill, and I. D. Russell. 1985. Evaluation of four disinfectants under poultry grow-out conditions using contact agar sampling technique. Poult. Sci. 64:629-633. <https://doi.org/10.3382/ps.0640629>

Gradel, K. O., and E. Rattenborg. 2003. A questionnaire-based, retrospective field study of persistence of *Salmonella* Enteritidis and *Salmonella* Typhimurium in Danish broiler houses. Prev. Vet. Med. 56:267-284. <https://doi.org/10.1016/s0167-5877(02)00211-8>

Jiang, L., M. Li, J. Tang, X. Zhao, J. Zhang, H. Zhu, X. Yu, Y. Li, T. Feng, and X. Zhang. 2018. Effect of different disinfectants on bacterial aerosol diversity in poultry houses. Front. Microbiol. 9:2113. <https://doi.org/10.3389/fmicb.2018.02113>

Kloska, F., M. Casteel, F. Wilms-Schulze Kump, and G. Klein. 2017b. Implementation of a risk-orientated hygiene analysis for the control of *Salmonella* JAVA in the broiler production. Curr. Microbiol. 74:356-364. <https://doi.org/10.1007/s00284-017-1199-9>

Le Bouquin, S., V. Allain, S. Rouxel, I. Petetin, M. Picherot, V. Michel, and M. Chemaly. 2010. Prevalence and risk factors for *Salmonella* spp. contamination in French broiler-chicken flocks at the end of the rearing period. Prev. Vet. Med. 97:245-251. <https://doi.org/10.1016/j.prevetmed.2010.09.014>

Li, M., J. Zhang, X. Zhang, J. Tang, M. Li, L. Jiang, X. Yu, and H. Zhu. 2019. Bacterial communities in PM2.5 and PM10 inside the cage broiler houses before and after disinfection. Iran. J. Vet. Res. 20:277-282. https://doi.org/10.22099/ijvr.2019.5505

Mageto, L. M., J. N. Ombui, and F. K. Mutua. 2018. Prevalence and risk factors for *Campylobacter* infection of chicken in peri-urban areas of Nairobi, Kenya. J. Dairy Vet. Anim. Res. 7:00184. <https://doi.org/10.15406/jdvar.2018.07.00184>

Marin, C., A. Hernandiz, and M. Lainez. 2009. Biofilm development capacity of *Salmonella* strains isolated in poultry risk factors and their resistance against disinfectants. Poult. Sci. 88:424-431. <https://doi.org/10.3382/ps.2008-00241>

Mo, S. S., A. B. Kristoffersen, M. Sunde, A. Nødtvedt, and M. Norström. 2016. Risk factors for occurrence of cephalosporin-resistant *Escherichia coli* in Norwegian broiler flocks. Prev. Vet. Med. 130:112-118. <https://doi.org/10.1016/j.prevetmed.2016.06.011>

Nasr, S. A. E. 2014. Application of foam mixture in disinfecting *Clostridium perfringens* isolated form broiler poultry litter. Glob. Vet. 13:273-277. <https://doi.org/10.5829/idosi.gv.2014.13.02.8528>

Newton, K., S. M. Withenshaw, S. A. Cawthraw, and R. Davies. 2021. In-depth farm investigations and an exploratory risk factor analysis for the presence of *Salmonella* on broiler farms in Great Britain. Prev. Vet. Med. 197:105498. <https://doi.org/10.1016/j.prevetmed.2021.105498>

Rose, N., F. Beaudeau, P. Drouin, J. Y. Toux, V. Rose, and P. Colin. 2000. Risk factors for *Salmonella* persistence after cleansing and disinfection in French broiler-chicken houses. Prev. Vet. Med. 44:9-20. <https://doi.org/10.1016/s0167-5877(00)00100-8>

Rose, N., J. P. Mariani, P. Drouin, J. Y. Toux, V. Rose, and P. Colin. 2003. A decision-support system for *Salmonella* in broiler-chicken flocks. Prev. Vet. Med. 59:27-42. <https://doi.org/10.1016/s0167-5877(03)00056-4>

Singer, R. S., J. S. Jeffrey, T. E. Carpenter, C. L. Cooke, E. R. Atwill, W. O. Johnson., and D. C. Hirsh. 2000. Persistence of cellulitis-associated *Escherichia coli* DNA fingerprints in successive broiler chicken flocks. Vet. Microbiol. 75:59-71. <https://doi.org/10.1016/s0378-1135(00)00205-4>

Ursinitsch, B., P. Pless, and J. Köfer. 2005. Prevalence and epidemiology of *Campylobacter* spp. in Styrian broiler herds. Vet. Med. Austria 92:93-99.

Vasiu, A., M. Niculae, E. Pall, and M. Spînu. 2014. The potential zoonotic risk due to cloacal flora in intensively raised broilers. Scientific Works. Series C. Veterinary Medicine, Vol. LX (1), ISSN 2065-1295, Pages 62-65.
